# Hidden Lineage Complexity of Glycan-Dependent HIV-1 Broadly Neutralizing Antibodies Uncovered by Digital Panning and Native-Like gp140 Trimer

**DOI:** 10.3389/fimmu.2017.01025

**Published:** 2017-08-24

**Authors:** Linling He, Xiaohe Lin, Natalia de Val, Karen L. Saye-Francisco, Colin J. Mann, Ryan Augst, Charles D. Morris, Parisa Azadnia, Bin Zhou, Devin Sok, Gabriel Ozorowski, Andrew B. Ward, Dennis R. Burton, Jiang Zhu

**Affiliations:** ^1^Department of Immunology and Microbial Science, The Scripps Research Institute, La Jolla, CA, United States; ^2^Department of Integrative Structural and Computational Biology, The Scripps Research Institute, La Jolla, CA, United States; ^3^International AIDS Vaccine Initiative Neutralizing Antibody Center and the Collaboration for AIDS Vaccine Discovery, The Scripps Research Institute, La Jolla, CA, United States; ^4^Scripps Center for HIV/AIDS Vaccine Immunology and Immunogen Discovery, The Scripps Research Institute, La Jolla, CA, United States; ^5^Department of Chemistry, The Scripps Research Institute, La Jolla, CA, United States; ^6^Ragon Institute of Massachusetts General Hospital, Massachusetts Institute of Technology, Cambridge, MA, United States

**Keywords:** antibody phage display, B-cell lineage development, broadly neutralizing antibodies, HIV-1 vaccine design, native-like trimer, next-generation sequencing

## Abstract

Germline precursors and intermediates of broadly neutralizing antibodies (bNAbs) are essential to the understanding of humoral response to HIV-1 infection and B-cell lineage vaccine design. Using a native-like gp140 trimer probe, we examined antibody libraries constructed from donor-17, the source of glycan-dependent PGT121-class bNAbs recognizing the N332 supersite on the HIV-1 envelope glycoprotein. To facilitate this analysis, a digital panning method was devised that combines biopanning of phage-displayed antibody libraries, 900 bp long-read next-generation sequencing, and heavy/light (H/L)-paired antibodyomics. In addition to single-chain variable fragments resembling the wild-type bNAbs, digital panning identified variants of PGT124 (a member of the PGT121 class) with a unique insertion in the heavy chain complementarity-determining region 1, as well as intermediates of PGT124 exhibiting notable affinity for the native-like trimer and broad HIV-1 neutralization. In a competition assay, these bNAb intermediates could effectively compete with mouse sera induced by a scaffolded BG505 gp140.681 trimer for the N332 supersite. Our study thus reveals previously unrecognized lineage complexity of the PGT121-class bNAbs and provides an array of library-derived bNAb intermediates for evaluation of immunogens containing the N332 supersite. Digital panning may prove to be a valuable tool in future studies of bNAb diversity and lineage development.

## Introduction

Broadly neutralizing antibodies (bNAbs) isolated from a small fraction of infected individuals have provided valuable insights into the humoral response against HIV-1 (1–4). It has been proposed that bNAbs with structurally defined antigen interactions can be used as templates for designing immunogens capable of eliciting similar antibody responses upon vaccination (5–7). Considering the extensive somatic hypermutation (SHM) and unusual sequence features of bNAbs, such as long complementarity-determining region (CDR) loops, an in-depth understanding of their ontogeny is imperative to designing sequential immunogens for guided antibody maturation (7). To this end, next-generation sequencing (NGS) has been utilized to explore details of the antibody repertoire and lineage development for bNAbs of vaccine interest (8–19). However, with the exception of a few cases (16, 17, 19–22), studies regarding early bNAb development continue to be restrained by limited sample availability and low frequency of lineage intermediates in memory B cells. Nonetheless, germline-reverted precursors and inferred lineage intermediates have been derived for several bNAbs targeting the CD4-binding site (CD4bs) and the N332 supersite near the base of variable loop 3 (V3) to facilitate immunogen design and *in vivo* evaluation (18, 23–31).

The HIV-1 envelope glycoprotein (Env) is covered with a dense layer of glycans. While this “glycan shield” poses barriers for bNAbs to access epitopes such as the CD4bs (17), it harbors key neutralizing antibody targets (32, 33) including the trimeric apex and the N332 supersite, of which the latter is a high-mannose patch centered around the N332 glycan (34, 35). The N332-dependent bNAb classes represented by PGT121, PGT128, and PGT135 have been extensively studied, showing an inherent promiscuity of the N332 supersite (36–42). NGS has revealed sequence diversity within the PGT121 and PGT135 families (9, 15, 18), with putative intermediates inferred for the former by phylogenetic analysis (18). The PGT121 class consists of bNAbs PGT121-123, PGT124/10-1074 (36), and PGT133-134, with PGT121 and 10-1074 demonstrating therapeutic potential in macaques and humans, respectively (43, 44). Structures of the PGT121-class bNAbs and their intermediates in complex with a modified gp120 core and BG505 SOSIP.664 gp140 trimer have placed PGT124 (and 10-1074 (36)) on a distinct evolutionary branch mainly focusing on the N332 glycan, whereas PGT121-123 recruited multiple glycans during lineage maturation (41, 42, 45). Recently, Steichen et al. designed a series of SOSIP.664 trimers with optimized binding to the PGT121 precursor and inferred intermediates (31), which induced bNAb-like responses in immunoglobulin (Ig) knock-in mice (23). This study provided a proof of concept for the B-cell lineage vaccine design targeting the N332 supersite (7). Furthermore, a ferritin nanoparticle displaying 24 copies of an N332 epitope-scaffold was reported to elicit a consistent N332-specific antibody response in BALB/c mice cross-reactive with a native-like trimer, which itself failed to induce such antibody response in immunization (46). Interestingly, once the membrane-proximal external region (MPER) and a C-terminal scaffold were included in this trimer construct, a robust antibody response to the apex was observed, indicating enhanced immune recognition of the glycan shield (46). Notwithstanding, several critical issues remain elusive in vaccine design targeting the N332 supersite: (i) whether native precursors and intermediates of the PGT121 class can be found within the donor repertoire; (ii) the minimal level of SHM required for the PGT121-class bNAbs to recognize an unmutated trimer; and (iii) whether a trimer immunogen with an intact glycan shield is capable of eliciting an N332-specific antibody response in animal immunization. A careful assessment of these issues is pertinent to future vaccine design efforts targeting the N332 glycan supersite.

In this study, we examined samples from donor-17, the source of PGT121-class bNAbs, to search for bNAb precursors and lineage intermediates. To facilitate this analysis, we developed a digital panning method by combining 900 bp NGS and H/L-paired antibodyomics with phage display of single-chain variable fragments (scFvs) to probe the donor repertoire. A biotinylated Avi-tagged BG505 gp140 trimer containing an optimized heptad repeat 1 (HR1) bend (47) was utilized as antigenic bait for antibody library screening. Although scFvs identified from this library may not possess authentic heavy and light chains, they nonetheless provide a glimpse into Env recognition by the diverse antibody repertoire that gave rise to the PGT121 lineage. Digital panning of a diverse scFv library identified bNAb-like clones with increased affinity for the native-like trimer, and PGT124 variants with a unique 2-aa insertion in the heavy chain complementarity-determining region 1 (HCDR1) loop. Similar sequences were also found in the donor antibody repertoire by deep sequencing, albeit with low frequency. A focused scFv library was then constructed using the PGT121 class-specific primers and subjected to digital panning against the trimer probe, revealing heavy and light chain (HC and LC) intermediates of the PGT121 class that differed notably from the inferred sequences (18). All library-derived antibody clones were assessed for antigen binding and HIV-1 neutralization. The utility of selected bNAb intermediates was further demonstrated using mouse sera from a previous trimer immunization (46). Serum analysis indicated that a scaffolded BG505 gp140.681 trimer induced consistent antibody responses to the apex and the N332 supersite, the latter of which could be blocked effectively by mature PGT124 and partially by a near-germline HC intermediate. Collectively, our study uncovers previously unrecognized lineage complexity of the PGT121-class bNAbs and presents a set of functional intermediates potentially valuable for the rational design and evaluation of HIV-1 immunogens containing the N332 supersite.

## Materials and Methods

### Human Specimen

Peripheral blood mononuclear cells (PBMCs) from an HIV-1 infected donor (donor-17) (34) of the Protocol G cohort were used for scFv library construction and antibody repertoire sequencing.

### HIV-1 Panning Antigens

The Avi-tagged BG505 gp140 trimer containing a redesigned HR1 bend (47) and the clade-C (ZM109) V1V2 nanoparticle (48) were transiently expressed in HEK293 F cells and in *N*-acetylglucosaminyltransferase I-negative (GnTI^−/−^) HEK293 S cells (Life Technologies), respectively. In brief, HEK293 F/S cells were thawed and incubated with FreeStyle™ 293 Expression Medium (Life Technologies) in a Shaker incubator at 37°C, with 120 rpm and 8% CO_2_. When cells reached a density of 2.0 × 10^6^/ml, expression medium was added to reduce cell density to 1.0 × 10^6^/ml for transfection with polyethylenimine (PEI-MAX) (Polysciences). For 1-l transfection of the gp140 trimer, 800 μg of plasmid, 300 μg of furin plasmid, and 300 μg of pAdVAntage were mixed in 25 ml of Opti-MEM transfection medium (Life Technologies) and added to 25 ml of Opti-MEM with 5 ml of PEI-MAX (1.0 mg/ml). For 1-l transfection of the V1V2 nanoparticle, 900 μg of plasmid was added to 25 ml of Opti-MEM and then mixed with 5 ml of PEI-MAX in 25 ml of Opti-MEM. After incubation for 30 min, the DNA-PEI-MAX complex was added to the cells. Culture supernatants were harvested 5 days after transfection, clarified by centrifugation at 1,800 rpm for 22 min, and filtered using 0.45 μm filters (Millipore). A *Galanthus nivalis* lectin (GNL) column (Vector Labs) was used to extract HIV-1 antigens from the supernatants and eluted with PBS containing 500 mM NaCl and 1 M methyl-α-d-mannopyranoside. For the Avi-tagged gp140 trimer, biotinylation was performed using the BirA biotin-protein ligase standard reaction kit (BirA-500) following the manufacturer’s instructions (Avidity). The gp140 trimer and the V1V2 nanoparticle were then purified using size-exclusion chromatography (SEC) on a HiLoad 16/600 Superdex 200 PG column and a Superose 6 10/300 GL column (GE Healthcare), respectively.

### Negative-Stain Electron Microscopy (EM)

The biotinylated Avi-tagged BG505 gp140 trimer, termed gp140.664.R1-Avi-Biot, was analyzed by negative-stain EM using a previously published protocol (47). Briefly, images were acquired with a Tietz 4 k × 4 k TemCam-F416 CMOS camera using a nominal defocus of 1,000 nm and the Leginon package (49) with an electron dose of ~29 e^−^/Å^2^. For image data processing, the Appion software package (50) was used to pick up particles and to make a stack. 2D classes were obtained using iterative multivariate statistical analysis (MSA)/multireference alignment (MRA) (51). To assess the quality of the trimers (native-like closed and open, or non-native), the reference-free 2D class averages were examined by eye using the same metrics as previously described (47). The 3D reconstruction of the gp140.664.R1-Avi-Biot trimer was obtained from the refinement of 17,783 particles using EMAN2 (52). The crystal structure of the BG505 SOSIP trimer (PDB ID: 4TVP) was fitted into the EM density and refined by using the UCSF Chimera “Fit in map” function (53).

### Biolayer Interferometry (BLI)

Antibody-binding kinetics of the biotinylated gp140 trimer was assessed using an Octet RED96 instrument (fortéBio) as previously described (47). All assays were performed with agitation set to 1,000 rpm in fortéBio 1× kinetic buffer. The final volume for all the solutions was 200 μl/well. Assays were performed at 30°C in solid black 96-well plates (Geiger Bio-One). 5 μg/ml of protein in 1× kinetic buffer was used to load the HIV-1 antibody on the surface of anti-human Fc Capture Biosensors (AHC) for 300 s. Typical capture levels were between 0.5 and 1 nm and variability within a row of eight tips did not exceed 0.1 nm. A 60-s biosensor baseline step was applied prior to the analysis of the association of the antibody on the biosensor to the trimer in solution for 200 s. A twofold concentration gradient of trimer starting at a maximum of 200 nM was used in a titration series of six. The dissociation of the interaction was followed for 300 s. Correction of baseline drift was performed by subtracting the mean value of shifts recorded for a sensor loaded with antibody but not incubated with trimer and for a sensor without antibody but incubated with trimer. Octet data were processed by fortéBio’s data acquisition software v.8.1. For apex-directed bNAbs, experimental data were fitted with the binding equations describing a 1:1 interaction, whereas for other bNAbs, the binding equations describing a 2:1 interaction were utilized to obtain the optimal fitting. *K*_D_ values were determined using the estimated response at equilibrium for each trimer concentration rather than the *k*_on_ and *k*_off_ values.

### Antibody Phage Display

The construction of scFv libraries was performed using a protocol modified from a previously described method (54). Briefly, total RNA was extracted from ~10 million PBMCs for single-stranded cDNA synthesis using the SuperScript™ III system (Life Technologies) with random hexamer and oligo(dT)12–18 primers. Antibody HC and LC variable regions were obtained from a primary polymerase chain reaction (PCR) with mixed HuJ reverse primers and separate HuV forward primers, including a set of forward primers designed to capture PGT121-class light chains containing framework region 1 (FR1) deletions (Table S1 in Supplementary Material). To generate HC-LC fragments, overlap PCR was performed in 25 × 50 μl reactions (10 cycles) with 50 ng of gel-purified HC and 50 ng of gel-purified LC (λ only or equal amounts of κ and λ LC) without primer. To obtain full-length scFv inserts, PCR was performed in 50 × 50 μl reactions (15 cycles) with S*fi*I-F and S*fi*I-R primers (Table S1 in Supplementary Material) using 100 ng of gel-purified HC-LC as template. The resulting scFv inserts and the phagemid vector, pAdL^TM^-20c (Antibody Design Labs), were digested with S*fi*I and gel-purified. The scFv DNA (256 ng) was ligated into the phagemid vector (400 ng) using the T4 Ligase Kit (New England BioLabs) in 25 × 40 μl reactions at 16°C overnight. Purified phagemids were electroporated into competent TG1 cells (Lucigen) with the MicroPulser™ system (Bio-Rad). Specifically, 1 μl of phagemids and 25 μl of competent cells were placed into a 0.1-cm cuvette for electroporation using the pre-set program at 1.8 kV. The transformed bacteria were spread on 2YT agar plates supplied with 100 μg/ml carbenicillin and 2% (w/v) glucose, which were incubated at 37°C overnight. Bacteria were then scraped from the plates for phage culture with the helper phage, CM13 (Antibody Design Labs), and biopanning. To facilitate NGS analysis, 3 ml of the transformed bacteria was grown in 50 ml 2YT-Carb-Glu medium at 37°C for 2 h, with the plasmids extracted using the Plasmid Midi Kit (Qiagen). Prior to biopanning, HIV-1 antigens were conjugated to magnetic beads following the manufacturer’s instructions (Invitrogen), with Dynabeads™ M-280 Streptavidin beads used for biotinylated gp140 trimers and Dynabeads™ M-270 Epoxy beads used for V1V2 nanoparticles. Four biopanning cycles were performed, with 6–8 wash steps in each cycle to remove phage that did not recognize the antigen. Plasmids were extracted from 3 ml of the bacteria after each cycle for subsequent NGS analysis of scFv libraries.

### Next-Generation Sequencing

Next-generation sequencing was performed on the Ion Torrent Personal Genome Machine (PGM) and S5 systems. The scFv-coding regions were amplified from the plasmid stock using PCR with fp1-S*fi*I-F and A-S*fi*I-[Barcode]-R primers (Table S1 in Supplementary Material). Of note, the forward primer (fp1-S*fi*I-F) contained a PGM full-length P1 (fP1) adaptor, whereas the reverse primer (A-S*fi*I-[Barcode]-R) contained a PGM A adaptor and an Ion Xpress™ barcode (Life Technologies) to differentiate each scFv library. A total of 25 PCR cycles were performed and the PCR products with an expected length of 800–900 bp were gel-purified (Qiagen). The procedure used for PGM sequencing has been described previously (20). Briefly, the libraries were quantitated using Qubit^®^ 2.0 Fluorometer with Qubit^®^ dsDNA HS Assay Kit. The dilution factor required for PGM template preparation was determined such that the final concentration was 50 pM. Template preparation was performed with the Ion PGM Template IA 500 Kit. Long-read sequencing was performed on the Ion Torrent PGM sequencer with the Ion PGM™ Hi-Q™ Sequencing Kit using an Ion 314 v2 chip for a total of 1,500 nucleotide flows. Raw sequencing data were processed without the 3′-end trimming in base calling to obtain full-length scFvs. The donor-17 HC library was generated using a 5′-RACE PCR protocol as previously described (17). Template preparation and (Ion 530) chip loading were performed on Ion Chef using the Ion 530 Ext Kit, followed by sequencing on the Ion S5 system with default settings. The raw NGS data can be found in the NCBI Sequence Read Archive with the accession number SRP105512.

### Bioinformatics Analysis of scFv Libraries

The human *Antibodyomics* 1.0 pipeline (8, 15, 17, 55) has been adapted to analyze the 900 bp sequencing data of scFv libraries. Following the housekeeping step (assigning a unique index to each scFv) and light-chain germline gene assignment, each scFv sequence was divided into HC and LC by matching a 15-aa (G_4_S)_3_ linker connecting the two chains. The HC and LC datasets were then processed separately by the chain-specific antibodyomics pipelines. Finally, full-length HC and LC from the same scFv were re-matched according to their indices in the sequenced scFv library (scFv indices) and deposited into correlated HC and LC databases for more in-depth analysis.

The *Antibodyomics* 1.0 pipeline consists of the following five steps (8, 15, 17): (1) data reformatting and cleaning; (2) germline gene assignment; (3) sequencing error correction; (4) calculation of sequence identity to a set of known antibodies; and (5) determination of CDR3 sequences and variable domain boundaries. In this study, a number of changes have been made to improve the pipeline accuracy and efficiency. In step 2, the original pipeline assigned variable (V), diversity (D), and joining (J) genes sequentially for heavy chains, whereas the modified pipeline first assigned V and J genes and then determined an appropriate D gene for the region between the V and J gene segments. In step 3, BLASTn (56) was replaced with LALIGN (57) to generate pairwise local alignment, as BLASTn often outputs a partial alignment lacking the N-terminus when sequencing errors occur in this region. By contrast, LALIGN can generate a more complete alignment with the assigned germline V gene, thus enabling error correction for the N-terminus and proper translation to the protein sequence. Of note, two consecutive deletions or insertions separated by a single nucleotide may produce a merged gap in alignment due to a lower gap penalty. Such homopolymer errors can now be detected and corrected by the modified pipeline. In step 5, CLUSTALW2 (58) was replaced by MUSCLE (59) to generate multiple sequence alignment, with a reduction of computational time by threefold to fourfold. The modified pipeline (version 2.0) has been validated using the 454 sequencing data reported for donor-17 (18). The *Antibodyomics* 2.0 pipeline can be obtained upon request to the authors.

### H/L-Paired, CDR3-Based Clustering Analysis

A novel method has been devised to determine non-redundant scFv clones and their frequencies within a converged phage library to facilitate antibody selection. This method is hierarchical, as it identifies the unique HCDR3 lineages first, and subsequently all the unique LCDR3 lineages associated with each HCDR3 lineage, resulting in a list of scFv clones each characterized by a distinct HCDR3-LCDR3 pair. In the first step, the unique HCDR3 lineages can be identified as follows: (1) HCDR3 loops are extracted from HC sequences and clustered into “groups” using CD-HIT (60) with an identity cutoff of 95% and a criterion of ≤1 mismatch within the core alignment; (2) a consensus HCDR3 is derived for each HCDR3 group based on the multiple sequence alignment by MUSCLE; and (3) HCDR3 groups are merged into unique HCDR3 “lineages” by BLASTClust (56) with a sequence identity of 95% or greater covering 95% of the sequence length. For each HCDR3 lineage, a similar procedure can be applied to the matching LCs to determine the unique LCDR3 lineages. The resulting scFv clones will be ranked by their frequencies (the number of scFvs possessing a pair of unique HCDR3 and LCDR3), with the HC and LC sequence files pertaining to each scFv clone provided as the output. For large scFv clonal families (>500 members), which potentially represent high-affinity binders in the enriched library, an additional clustering analysis is performed to derive high-quality consensus sequences for antibody synthesis and functional validation. In brief, the H/LCDR3 sequences divisible by 3 will be clustered into groups using CD-HIT with an identity cutoff of 95%, no mismatch within the core alignment and identical sequence lengths. The largest group is then manually inspected and subjected to the consensus calculation by MUSCLE using no more than 4,000 randomly selected sequences. For small scFv clonal families (≤500 members), all sequences in the dataset are used to derive consensus HC and LC. The program used to perform H/L-paired, CDR3-based clustering analyses can be obtained upon request to the authors.

### Enzyme-Linked Immunosorbent Assay (ELISA)

Each well of a Costar™ 96-well assay plate (Corning) was first coated with 50 μl PBS containing 0.2 μg of the appropriate antigens. The plates were incubated overnight at 4°C and, then, washed five times with wash buffer containing PBS and 0.05% (v/v) Tween 20. Each well was then coated with 150 μl of a blocking buffer consisting of PBS, 20 mg/ml blotting-grade blocker (Bio-Rad), and 5% (v/v) FBS. The plates were incubated with the blocking buffer for 1 h at room temperature and, then, washed five times with wash buffer. Wild-type (WT) PGT121-class bNAbs and antibodies derived from the donor-17 scFv library were diluted in the blocking buffer to a maximum concentration of 1 or 10 μg/ml, followed by a 10-fold dilution series. For each antibody dilution, a total of 50 μl volume was added to the appropriate wells. Each plate was incubated for 1 h at room temperature and, then, washed five times with wash buffer. A 1:5,000 dilution of goat anti-human IgG antibody (Jackson ImmunoResearch Laboratories, Inc.) was then made in the wash buffer, with 50 μl of this diluted secondary antibody added to each well. The plates were incubated with the secondary antibody for 1 h at room temperature and, then, washed five times with wash buffer. Finally, the wells were developed with 50 μl of TMB (Life Sciences) for 3–5 min before stopping the reaction with 50 μl of 2 N sulfuric acid. The resulting plate readouts were measured at a wavelength of 450 nm.

### HIV-1 Neutralization

Neutralization assays were performed on TZM-bl reporter cells using a panel of six tier-2 isolates and two tier-1 isolates. Neutralization curves were fit by a non-linear regression analysis using a 5-parameter hill slope equation. The 50% inhibitory concentration (IC_50_) is defined as the antibody concentration required for inhibiting HIV-1 infection by 50%.

### Serum Binding and Competition ELISA

Mouse antisera from a previous trimer immunization were tested against a native-like BG505 gp140 trimer (gp140.664.R1), a V1V2 nanoparticle (V1V2-FR), and an N332 nanoparticle (1GUT_A_ES-FR) by ELISA following a previously described protocol (46). Competition ELISA was performed to measure the binding of mouse antisera elicited by a scaffolded gp140 trimer to an N332 nanoparticle in the presence of WT bNAbs or native intermediates (NINs) derived from a focused donor-17 library. A slightly modified protocol was used. Briefly, plates were coated with purified N332 nanoparticles at 0.2 μg per well and incubated overnight at 4°C. After blocking, 10–50 μg/ml of a bNAb variant was added to the plates at 50 μl per well for 1 h incubation. The mouse antisera were initially diluted by a factor of 5 in blocking buffer, followed by a 10-fold dilution series. A 50 μl volume of each dilution was then added to the wells without washing the plates. After incubation for 1 h and five washes, a 1:2,000 dilution of goat anti-mouse IgG antibody was added to each well at 50 μl per well, followed by development with 50 μl of TMB, stop with 50 μl of 2 N sulfuric acid, and measurement at 450 nm.

## Results

### A Native-Like gp140 Trimer Probe for Identification of Env-Specific Antibodies

The HIV-1 Env spike, a trimer of gp120 and gp41 heterodimers, is the only target of neutralizing humoral immune response (61). Trimer-based HIV-1 vaccine design has long been hampered by the metastable nature of the Env. A cleaved, soluble BG505 SOSIP.664 gp140 trimer was recently developed that closely mimics the functional Env spike in the stable, prefusion conformation (62–68). The BG505 SOSIP.664 trimer has enabled high-resolution structural analysis of the Env spike in complex with many bNAbs by crystallography and EM (42, 69–75). Alternative trimer platforms have also been developed, including the native, flexibly linked trimer (76–78), the single-chain gp140 (sc-gp140) trimer (79), and the uncleaved, prefusion-optimized (UFO) trimer (47). In particular, the UFO design has demonstrated greater trimer yield and purity for diverse HIV-1 strains in comparison to the SOSIP design (47). Furthermore, a cleaved version of the UFO trimer has been displayed on various nanoparticles as multivalent immunogens (48).

**Figure 1:**
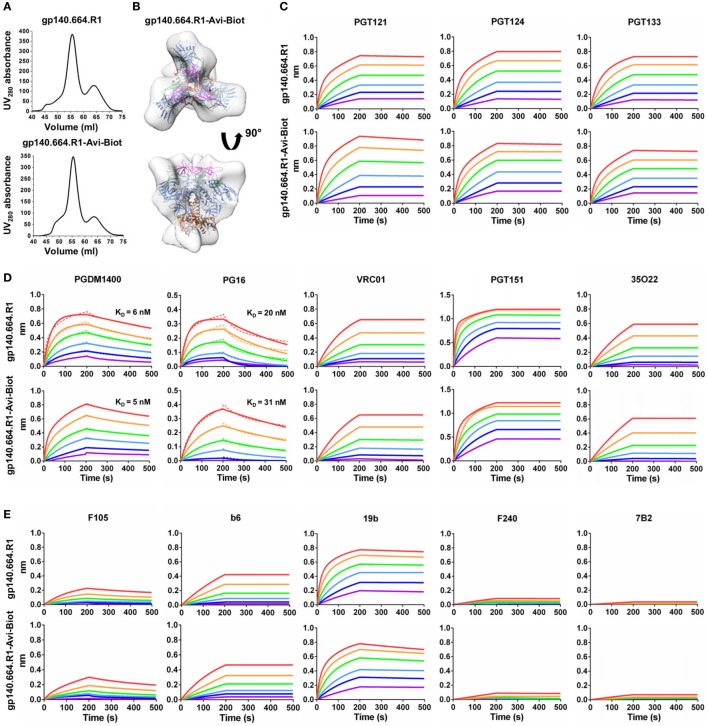
Biophysical characterization of a native-like, prefusion-optimized trimer probe. This trimer probe was designed based on a BG505 gp140 construct containing a redesigned heptad repeat 1 bend (termed gp140.664.R1). **(A)** Size-exclusion chromatography (SEC) profiles of 293F-expressed, *Galanthus nivalis* lectin (GNL)-purified gp140.664.R1 trimer (top) and biotinylated, Avi-tagged gp140.664.R1 trimer probe, termed gp140.664.R1-Avi-Biot (bottom), from a Superdex 200 16/600 column. **(B)** 3D reconstruction of the gp140.664.R1-Avi-Biot trimer probe derived from negative-stain electron microscopy (EM). The trimer densities are shown in gray transparent surface with the fitted crystal structure of the SOSIP trimer (PDB ID: 4TVP, gp120 in blue with V1V2 in magenta, V3 in green, and gp41 in brown). **(C–E)** Antigenic profiles of the gp140.664.R1 and gp140.664.R1-Avi-Biot trimers measured for **(C)** three broadly neutralizing antibodies (bNAbs) of the PGT121 class (PGT121, 124, and 133) isolated from donor-17, **(D)** five bNAbs targeting other sites of vulnerability on the HIV-1 envelope glycoprotein, and **(E)** five representative non-NAbs. Sensorgrams were obtained from an Octet RED96 instrument using a trimer titration series of six concentrations (200–6.25 nM by twofold dilution). *K*_D_ values calculated from 1:1 global fitting are labeled for V1V2 apex-directed bNAbs (PGDM1400 and PG16) in panel **(D)**.

Here, we designed a cleaved BG505 trimer probe for Env-specific antibody identification, which contains a redesigned HR1 bend—the basis of the UFO design (47)—and a C-terminal Avi-tag. This trimer probe was expressed transiently in HEK293 F cells with co-transfected furin as previously described (47). The secreted Env protein was purified using a GNL column followed by *in vitro* biotinylation and SEC on a Superdex 200 16/600 column. We first compared the SEC profiles of the tagged and untagged trimers based on the ultraviolet absorbance at 280 nm (Figure 1A). The biotinylated Avi-tagged trimer probe, termed gp140.664.R1-Avi-Biot, displayed high yield and purity on par with the untagged trimer. We then analyzed the trimer probe by negative-stain EM, which yielded a 3D reconstruction consistent with the previously reported structures of HR1-redesigned trimers and UFO trimers (Figure 1B; Figure S1 in Supplementary Material) (47). The unoccupied EM densities extending from the C-termini of the docked gp140 trimer model may correspond to the biotinylated Avi-tag. Using BLI, we assessed the antigenicity of the trimer probe against a panel of bNAbs and non-NAbs. Three bNAbs isolated from donor-17 (PGT121, 124, and 133) (34) were used to assess the recognition of the N332 supersite by the PGT121-class bNAbs and to establish a baseline for comparison with forthcoming antibodies identified from this donor (Figure 1C). Octet binding revealed similar kinetics for the trimer probe and the untagged trimer, showing fast on-rates and flat dissociation curves. Consistently, nearly identical kinetic profiles were observed for bNAbs targeting other conserved epitopes, including the apex recognized by PGDM1400 (80) and PG16 (35), the CD4bs by VRC01 (81), and the gp120-gp41 interface by PGT151 (82) and 35O22 (83) (Figure 1D). Five non-NAbs were utilized to assess the exposure of non-neutralizing epitopes on the Env surface (Figure 1E). While the trimer probe appeared to shield non-neutralizing epitopes to the same degree as the untagged parent trimer, both outperformed the SOSIP trimer (47).

Our results thus demonstrated that the addition of an Avi-tag and *in vitro* biotinylation had no adverse effect on trimer purity, structural integrity, or antigenicity. In recent studies, the BG505 SOSIP.664 trimer was used as a bait to isolate bNAbs from HIV-1 patient samples (84, 85). As one of the well-characterized trimer platforms (86, 87), the UFO trimer may provide an alternative probe to identify bNAbs of diverse specificities from antibody libraries or Env-specific B cells.

### Long-Read (900 bp) NGS and H/L-Paired Antibodyomics Enable Digital Panning

Phage display (88, 89) has been widely used to produce monoclonal antibodies (mAbs) for research and therapeutic purposes (90–94). While the first HIV-1 bNAbs were isolated from phage libraries (95), single-cell approaches have contributed most of the new bNAbs (96). Nonetheless, both scFvs and fragment antigen binding regions can be displayed on the phage surface and subjected to a selection process known as biopanning. Although NGS has been used to estimate the diversity of antibody phage libraries (97, 98), its broader application has been restricted by the insufficient read length to cover both HC and LC in a scFv sequence (>800 bp). It was recently proposed that once this technical barrier is overcome, NGS can be used to directly select functional mAbs from a scFv library (99, 100), rendering a quantitative method for antibody discovery.

**Figure 2:**
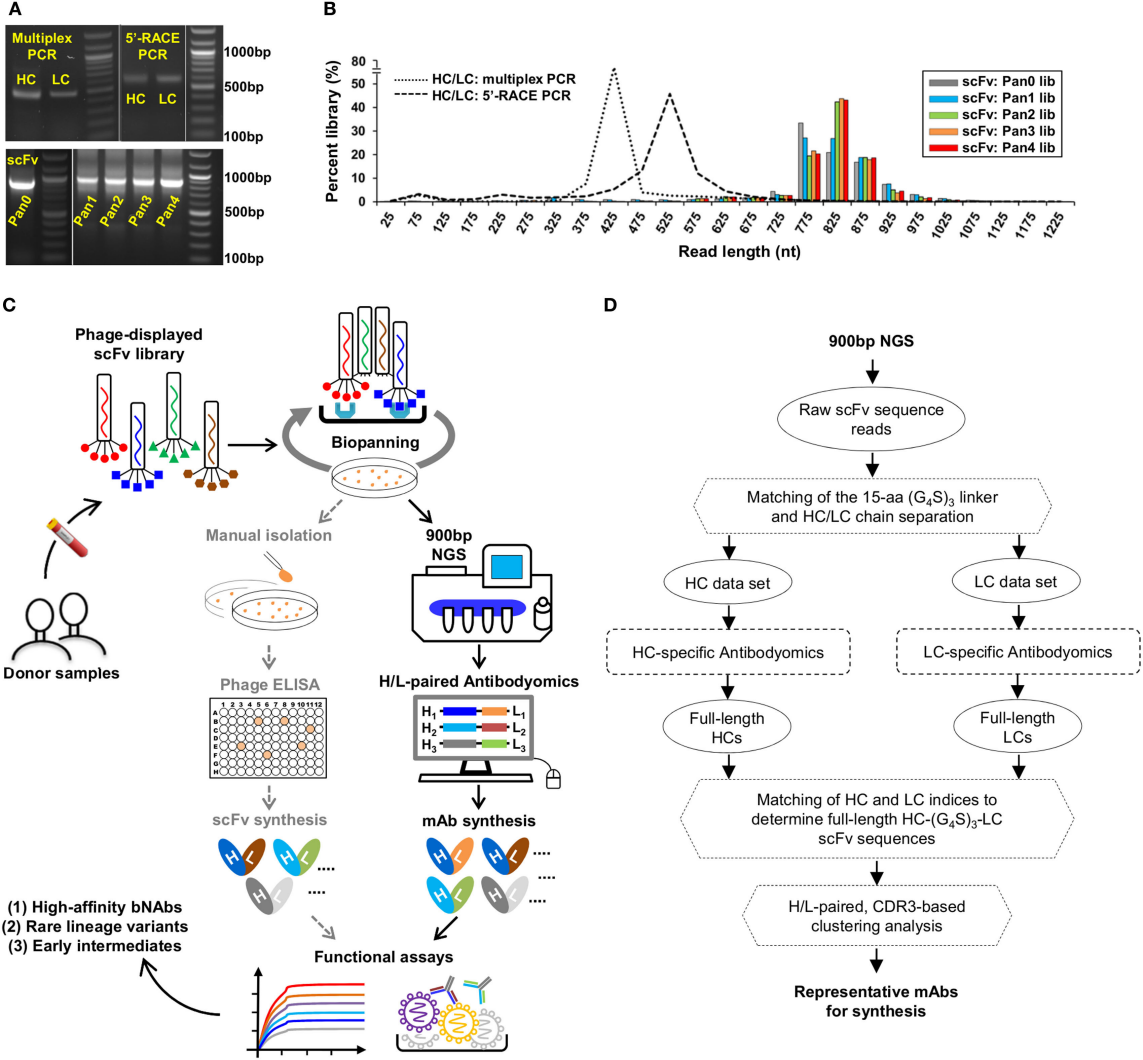
Digital panning, a quantitative method for identification of functional antibodies. **(A)** Gel electrophoresis of three donor-17 antibody libraries generated from multiplex PCR using gene-specific primers, 5′-RACE PCR using single 3′-reverse primers, and H/L-overlapping PCR using gene-specific primers. For the purpose of formatting, the DNA gel has been rearranged with splicing (labeled with white lines). **(B)** Read length distributions of three donor-17 antibody libraries obtained from Personal Genome Machine (PGM) sequencing. Five single-chain variable fragment (scFv) libraries are plotted as histograms and color-coded according to their antigen panning steps: gray (Pan0), cyan (Pan1), green (Pan2), orange (Pan3), and red (Pan4). The two mixed HC/LC libraries generated from multiplex PCR and 5′-RACE PCR are shown as black dotted and dashed lines, respectively. **(C)** Schematic view of the digital panning method with the route of conventional panning included for comparison. Briefly, a scFv library is first constructed from donor peripheral blood mononuclear cells (PBMCs) and displayed on the phage surface for biopanning against an optimized antigen. The pre- and post-panning scFv libraries are sequenced on PGM to achieve 900 bp read length, with the sequenced full-length scFv libraries processed and analyzed by an H/L-paired antibodyomics pipeline. Representative scFv clones are selected for mAb synthesis and functional characterization. In HIV-1 bNAb studies, digital panning may identify high-affinity bNAb-like scFvs, rare bNAb lineage variants, and early intermediates. **(D)** Schematic view of an H/L-paired antibodyomics analysis.

Here, we addressed this technical challenge in a case study of the PGT121 lineage. We first characterized three types of antibody libraries constructed from PBMCs of donor-17 using gel electrophoresis (Figure 2A). Two antibody libraries were generated using different PCR methods—multiplex PCR (with gene-specific primers) and 5′-RACE PCR (with single 3′-reverse primers)—and showed bands around 500 and 600 bp, respectively (15). By contrast, the scFv library generated using H/L-overlapping PCR and a large primer set (Table S1 in Supplementary Material), as well as four post-panning libraries, yielded distinctive bands around 900 bp. This comparison highlights the difficulty in sequencing full-length scFv libraries. Previously, we demonstrated an extended read length of 600–700 bp in the unbiased NGS analysis of antibody repertoires using the Ion Torrent PGM and a modified protocol with 1,200 nucleotide flows (15, 17, 46, 101). Can this NGS platform be adapted for sequencing scFvs? To test this possibility, we sequenced the donor-17 scFv libraries on the PGM using an Ion 314v2 chip and 1,500 nucleotide flows (Figure 2B). Markedly, over one million raw reads in the range of 750–950 bp were obtained for the scFv libraries, compared with 450 and 550 bp for the mixed HC/LC libraries generated by two different PCR methods.

We then combined 900 bp long-read NGS and H/L-paired antibodyomics with scFv library panning into a coherent strategy, termed digital panning, for identification of functional mAbs (Figure 2C). By design, this method is both analytical and deterministic, as it can capture the full-length scFvs during the panning process and select representative scFv clones based on their frequency and antibody characteristics. The previously developed *Antibodyomics* pipeline (55) was modified to facilitate *in silico* analysis of the sequenced scFv libraries (Figure 2D). Briefly, each scFv is assigned a unique index and divided into HC and LC by matching a 15-aa linker between the two chains. Following the chain-specific pipeline processing, HC and LC from the same scFv are identified based on their shared scFv indices, resulting in correlated HC and LC databases. Of note, the method used in the *Antibodyomics* pipeline for indel error correction (9) has been modified to achieve greater accuracy, as demonstrated for the 454 sequencing data from donor-17 (18) (Figure S2 in Supplementary Material). The scFv-derived HC and LC databases can then be analyzed in-depth using bioinformatics tools previously developed for repertoire profiling and lineage tracing (8–12, 15, 17). Further implementation of an H/L-paired, CDR3-based clustering method allows determination of representative scFv clones and their respective frequencies for facilitating mAb selection.

### Digital Panning of a Diverse Donor-17 ScFv Library Identifies a New Lineage Variant

As described earlier, a diverse donor-17 scFv library has been constructed using primers covering all HC and LC germline gene families (Table S1 in Supplementary Material) and screened against a native-like trimer probe (Figure 1). The pre-panning scFv library and four post-panning scFv libraries were pooled at a ratio of 3:1:1:1:1 for 900 bp deep sequencing on the PGM (Table S2A in Supplementary Material). After data processing with the H/L-paired *Antibodyomics* pipeline (Figure 2D), the scFv-derived HC and LC databases were analyzed to generate library profiles and to select Env-specific mAb clones.

**Figure 3:**
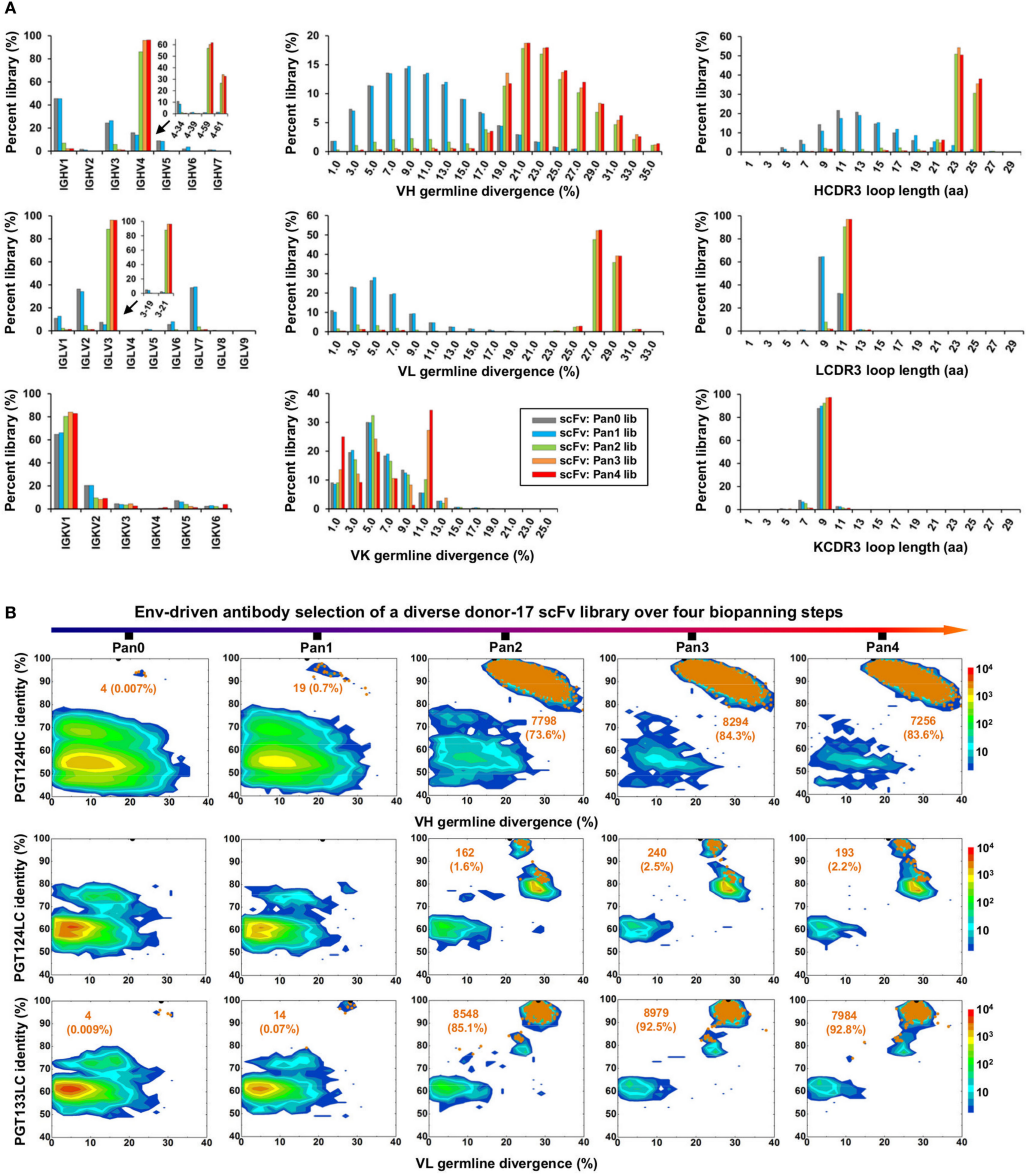

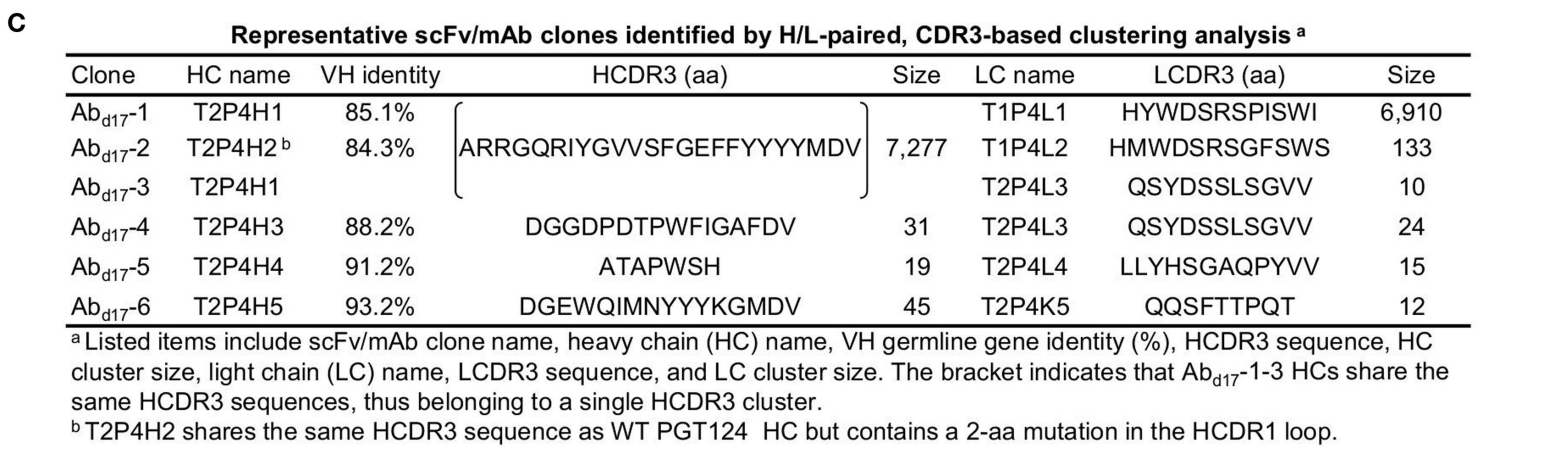
Digital panning of a diverse donor-17 single-chain variable fragment (scFv) library against the native-like trimer. A diverse scFv library was constructed from donor-17 peripheral blood mononuclear cells (PBMCs) using a complete set of primers. **(A)** Distributions of germline gene usage (left), somatic hypermutation (middle), and CDR3 loop length (right) are plotted for the five scFv libraries obtained from the trimer panning process. Distributions of prevalent germline gene families within IgHV4 and IgLV3 (>1%) are shown as insert images. Histograms are color-coded following the same scheme as in Figure 2B. **(B)** Identity-divergence analysis of the five donor-17 scFv libraries obtained from the trimer panning process. For each library, sequences are plotted as a function of sequence identity to PGT124 HC (top), PGT124 LC (middle), and PGT133 LC (bottom), and sequence divergence from germline genes. Color-coding indicates sequence density at a particular point on the 2D plot. Wild-type (WT) PGT124 and PGT133 are labeled on the 2D plots as black dots. Sequences with CDR3 identity of 95% or greater are shown as orange dots, with the number of sequences and library percentage labeled on the 2D plot for comparison. **(C)** Representative scFv clones identified from a diverse scFv library by H/L-paired, CDR3-based clustering analysis for mAb synthesis.

Quantitative library profiles displayed a distinct pattern of antibody enrichment and a rapid convergence after two panning steps. In terms of germline gene usage (Figure 3A, column 1), the IgHV4 and IgLV3 gene families accounted for >96% of the library upon convergence, with a 6–12-fold increase in frequency with respect to the pre-panning library. Further analysis revealed the prevalence of IgHV4-59 (~60%) and IgHV4-61 (~30%) within the IgHV4 family and IgLV3-21 (~96%) within the IgLV3 family. In contrast, the κ LCs exhibited a less discernible pattern of germline gene usage and a reduction to less than 100 sequences in the converged scFv library. A significant shift was observed in the SHM distribution (Figure 3A, column 2), with the average value increasing from 11% and 6% to 24% and 27% for HC and LC, respectively. Furthermore, nearly 90% of the HC sequences possessed long HCDR3 loops of 23–25 aa (Figure 3A, column 3). Overall, this scFv library appeared to have converged to the PGT121-class bNAbs, which are characterized by a specific germline gene usage (IgHV4-59 and IgLV3-21), high degree of SHM (HC: 19–22% and LC: 21–29%), and a long HCDR loop (24 aa). A two-dimensional (2D) identity-divergence analysis was then performed to visualize the scFv-derived HC and LC populations during the trimer panning process (Figure 3B). Indeed, the 2D plots revealed rapid enrichment of PGT124-like HCs and PGT124/PGT133-like LCs, which was further confirmed by the H/L-paired, CDR3-based clustering analysis (Figure 3C). While the most prevalent scFv family was characterized by PGT124-like HCDR3 loops, ~30% of HCs in this family were assigned to IgHV4-61 instead of IgHV4-59 due to a 2-aa insertion in HCDR1. Of note, the PGT133-like LCs appeared to be more prevalent than the PGT124-like LCs in the converged library, showing ~50-fold difference in their frequencies. In addition, a small group of 45 scFvs possessed a 16-aa HCDR3 loop partially matching the C-terminal portion of the PGT124 HCDR3 sequence. Taken together, digital panning of a diverse donor-17 scFv library resulted in a panel of mAbs including predominant bNAb-like clones and mAbs of unknown specificities.

**Figure 4:**
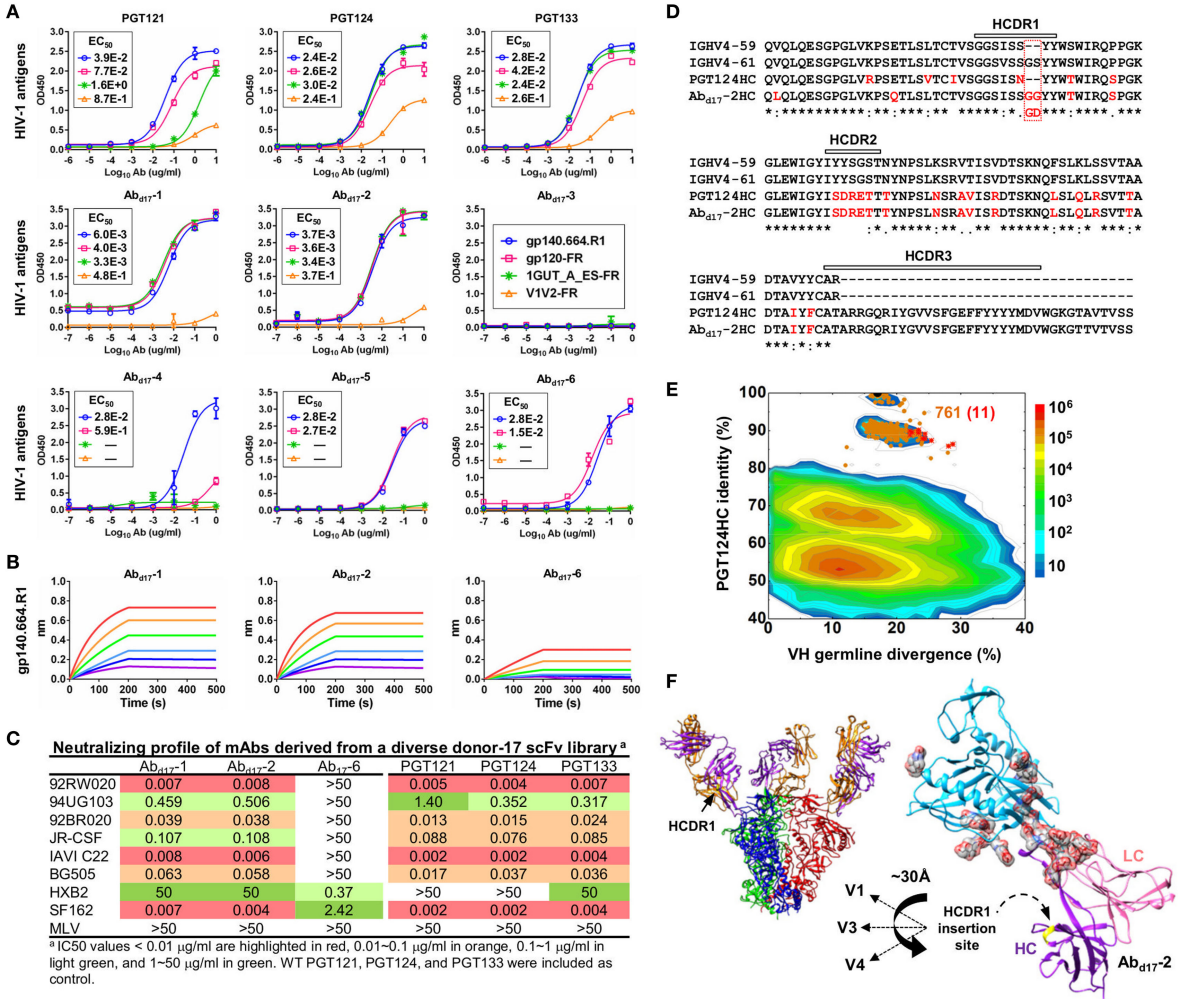
Characterization of monoclonal antibodies (mAbs) identified from a diverse donor-17 single-chain variable fragment (scFv) library. **(A)** Enzyme-linked immunosorbent assay (ELISA) binding of three broadly neutralizing antibodies (bNAbs) of the PGT121 class (PGT121, 124, and 133) and six scFv-derived mAbs, Ab_d17_-1–6, to four HIV-1 antigens including a native-like trimer (gp140.664.R1), a gp120 nanoparticle (gp120-FR), a nanoparticle presenting 24 copies of the scaffolded N332 supersite (1GUT_A_ES-FR), and a clade-C V1V2 nanoparticle (V1V2-FR). EC_50_ values are labeled for all ELISA plots except for instances in which the highest OD_450_ value is below 0.1 or in the cases of ambiguous data fitting. **(B)** Octet binding of Ab_d17_-1, -2, and -6 to the native-like trimer gp140.664.R1. Sensorgrams were obtained from an Octet RED96 instrument using a titration series of six concentrations (200–6.25 nM by twofold dilution). **(C)** Neutralization profiles of Ab_d17_-1, -2, and -6 against six tier-2 viruses and two tier-1 viruses, with WT bNAbs PGT121, 124, and 133 included as references. IC_50_ values are highlighted based on the following color-coding scheme: <0.01 μg/ml (red), 0.01–0.1 μg/ml (orange), 0.1–1 μg/ml (light green), and 1–50 μg/ml (green). **(D)** Sequence alignment of Ab_d17_-2 with two HC germline genes (IgHV4-59 and IgHV4-61) and PGT124 HC. The three HCDR regions are marked above the sequences, with the mutations with respect to IgHV4-59 colored in red. **(E)** Identity-divergence analysis of the HC repertoire obtained from S5 sequencing. Sequences are plotted as a function of sequence identity to PGT124 HC and sequence divergence from germline genes. Color-coding indicates sequence density at a particular point on the 2D plot. PGT124 HC is labeled on the 2D plot as a black dot. Sequences with HCDR3 identity of 95% or greater to PGT124 are shown as orange dots on the 2D plots, with the ones containing a 2-aa heavy chain complementarity-determining region 1 (HCDR1) insertion shown as red asterisks. **(F)** Structural models of Ab_d17_-2 in complex with a native-like trimer (gp140.664.R1) and engineered gp120 outer domain (eOD). The Ab_d17_-2:gp140 complex was modeled upon two trimer structures (PDB IDs: 5JS9 and 5T3S), while the Ab_d17_-2:eOD complex was modeled upon the PGT124:eOD complex (PDB ID: 4R2G). While the proteins are shown as ribbons in both cases, the molecular surface is also shown for glycans in the latter case. The distance (~30 Å) between the Ab_d17_-2 HCDR1 and three gp120 glycan-rich loops is labeled.

The six most prevalent mAbs (Ab_d17_-1–6) in the H/L-paired clustering analysis (Figure 3C) were synthesized for functional validation (Table S3A in Supplementary Material). Antigen binding was initially assessed by ELISA against a native-like trimer (gp140.664.R1) (47), a gp120-ferritin (gp120-FR) nanoparticle (48), and an N332 nanoparticle (1GUT_A_ES-FR) (46), with a V1V2-ferritin nanoparticle (V1V2-FR) (48) included as a negative control. Three representative bNAbs for the PGT121 class demonstrated differential binding profiles: PGT124 bound to the trimer and the N332 nanoparticle with comparable EC50s, whereas PGT121 showed preferential binding to the gp140 trimer (Figure 4A). This finding is consistent with the reports that PGT121 engages multiple glycans on the Env (41, 42), which may not all be included in the scaffolded N332 supersite on the nanoparticle surface (46). Among the library-derived mAbs, the two PGT124-like clones (Ab_d17_-1 and Ab_d17_-2) appeared to be the best performers, binding to the N332-containing antigens with EC50 values ~10-fold lower than the WT bNAbs in addition to the reduced V1V2 recognition. Among the other mAbs, Ab_d17_-5 and Ab_d17_-6 bound the native-like trimer and the gp120 nanoparticle but not the N332 nanoparticle, whereas Ab_d17_-3 and Ab_d17_-4 displayed poor antigen binding. The “YYY–MDV” segment in the Ab_d17_-6 HCDR3, which partially matches to the PGT124 HCDR3, might be involved in Env binding, but detailed structural characterization would be required to confirm this hypothesis. When evaluated by BLI (Figure 4B), Ab_d17_-1 and Ab_d17_-2 exhibited PGT124-like trimer binding profiles in comparison with a weak binding signal observed for Ab_d17_-6. HIV-1 neutralization was tested against a panel of six tier-2 viruses and two tier-1 viruses (Figure 4C). While Ab_d17_-1 and Ab_d17_-2 displayed the same neutralization breadth and potency as the WT bNAbs, Ab_17_-6 only neutralized two tier-1 viruses, indicating the presence of diverse NAb lineages, including the PGT121 lineage, in the donor repertoire.

A key finding thus far in the analysis of a diverse donor-17 scFv library was Ab_d17_-2, which represented PGT124-like clones with a putative IgHV4-61 origin. Sequence alignment of Ab_d17_-2 with IgHV1-59, IgHV4-61, and PGT124 revealed a unique 2-aa insertion preceding the “YY” motif in the HCDR1 loop (Figure 4D). Is this insertion biologically relevant or merely an error that occurred in the scFv library construction? To answer this question, we prepared an HC-only library from the donor-17 PBMCs (that were used to construct scFv libraries) with a previously reported 5′-RACE PCR protocol (15, 17, 46, 101) and sequenced this library on the Ion S5 platform using an Ion 530 chip. S5 sequencing yielded over 12 million raw reads, which were processed by the *Antibodyomics* 2.0 pipeline, resulting in 7.8 million full-length HCs for repertoire profiling and identity-divergence analysis (Figures S3A,B in Supplementary Material). HC populations with a PGT124 identity of 85–100% and a germline divergence of 15–25% were observed on the 2D plot (Figure 4E). Among the 761 sequences with an HCDR3 identity of 90% or greater to PGT124, 11 were assigned to IgHV4-61 with HCDR1 insertions. Interestingly, similar HCDR1 insertions were also found in PGT122 HC variants (Figure S3C in Supplementary Material). Ultra-deep repertoire sequencing thus provided evidence that this HCDR1 insertion was likely a result of lineage diversification. Structural modeling of Ab_d17_-2 in complex with a native-like gp140 trimer (47) and an engineered gp120 outer domain revealed potential roles of this HCDR1 insertion (Figure 4F). As previously reported, the PGT121-class bNAbs utilize an open face formed by three HCDRs to interact with various components of the Env (37, 41, 42). Due to the close proximity of HCDR1 to HCDR2, some Env interactions might be shifted to HCDR1 as a means to accommodate the rapidly changing glycan shield. Indeed, the HCDR1 insertion site is ~30 Å from the glycan-rich V1, V3, and V4 loops, suggesting that an extended loop at this site may mediate interactions with multiple gp120 glycans.

In summary, digital panning of a diverse donor-17 scFv library identified bNAb-like scFv clones and a previously unknown HCDR1 variation within the PGT121 class. For comparative analysis, we also screened this scFv library against a clade-C V1V2 nanoparticle (48), which did not yield any neutralizing mAbs to the apex (Tables S2B and S3B, and Figures S4 and S5 in Supplementary Material), in line with the previous findings for this patient (34). Therefore, our results demonstrated a focused antibody repertoire tuned for specific recognition of a glycan supersite on the native Env.

### Digital Panning of a Focused Donor-17 ScFv Library Identifies bNAb Intermediates

Previously, Sok et al. developed a novel phylogenetic method to derive putative intermediates of PGT121-134 based on the 454 sequencing data (18). These intermediates have been studied in detail to inform on the early events of bNAb development (41, 42) and to design trimer immunogens for sequential immunization of Ig knock-in mice (23, 31). Undoubtedly, patient samples will provide a more reliable source of bNAb precursors and intermediates, which, however, are only present at low frequencies within the memory B-cell repertoire. In this study, we hypothesized that digital panning of a bNAb-lineage-focused scFv library may capture these important but rare clones that would otherwise be inaccessible to the standard methods of antibody identification (96).

**Figure 5:**
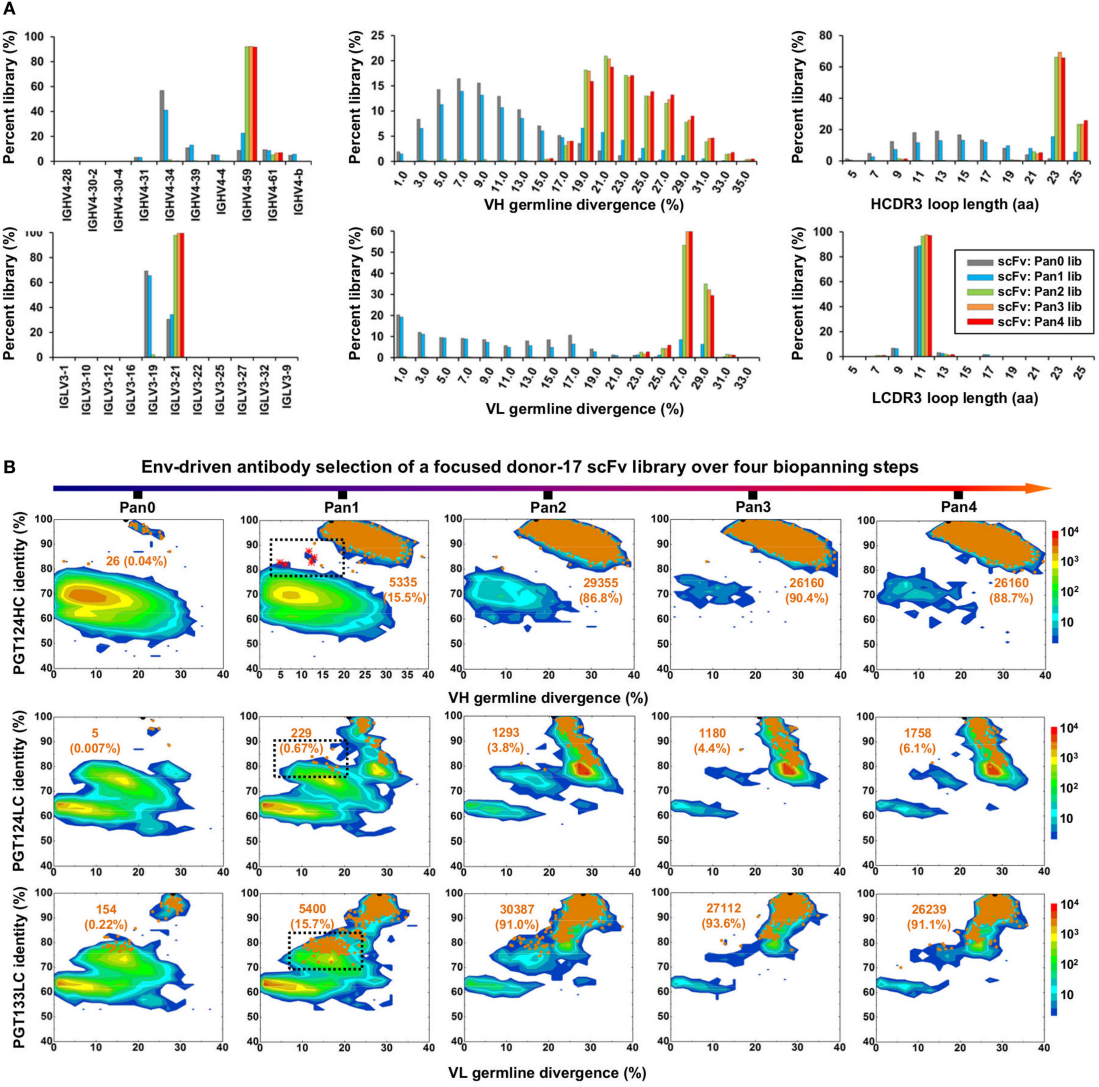
Digital panning of a focused donor-17 single-chain variable fragment (scFv) library against the native-like trimer. A focused scFv library was constructed from donor-17 peripheral blood mononuclear cells (PBMCs) using IgHV4- and IgLV3-specific primers. **(A)** Distributions of germline gene usage (left), somatic hypermutation (middle), and CDR3 loop length (right) are plotted for the five scFv libraries obtained from the trimer panning process. Histograms are color-coded following the same scheme as in Figure 2B. **(B)** Identity-divergence analysis of the five donor-17 scFv libraries obtained from the trimer panning process. For each library, sequences are plotted as a function of sequence identity to PGT124 HC (top), PGT124 LC (middle), and PGT133 LC (bottom), and sequence divergence from germline genes. Color-coding indicates sequence density at a particular point on the 2D plot. WT PGT124 and PGT133 are labeled on the 2D plots as black dots. Sequences with CDR3 identity of 95% or greater are shown as orange dots, with the number of sequences and library percentage labeled on the 2D plot for comparison. Sequences with low levels of SHM and moderate identity to the respective broadly neutralizing antibodies, corresponding to native intermediates (NINs), are circled by black dotted line boxes on the 2D plots for the Pan1 library. Five NIN-like scFv clones selected from the Pan1 library based on the HC identity to PGT124 are shown as red asterisks on the 2D plot.

**Figure 6:**
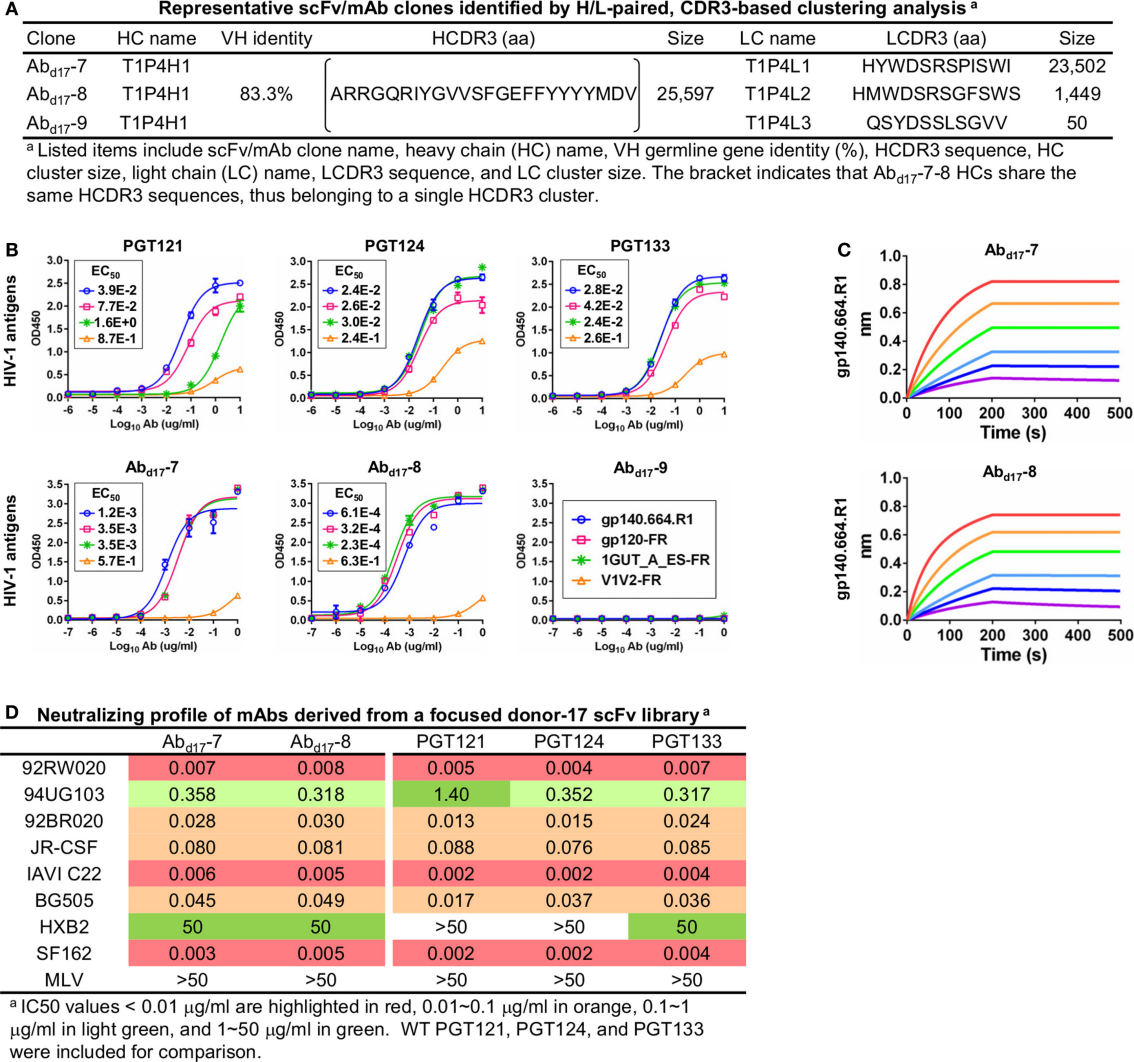
Characterization of monoclonal antibodies (mAbs) identified from a focused donor-17 single-chain variable fragment (scFv) library. **(A)** Representative scFv clones identified from a focused scFv library by H/L-paired, CDR3-based clustering analysis for mAb synthesis. **(B)** Enzyme-linked immunosorbent assay (ELISA) binding of three broadly neutralizing antibodies (bNAbs) of the PGT121 class (PGT121, 124, and 133) and three scFv-derived mAbs, Ab_d17_-7–9, to the gp140.664.R1 trimer and three nanoparticles (gp120-FR, 1GUT_A_ES-FR, and V1V2-FR). EC_50_ values are labeled for all ELISA plots except for instances in which the highest OD_450_ value is below 0.1 or in the cases of ambiguous data fitting. **(C)** Octet binding of Ab_d17_-7 and -8 to the gp140.664.R1 trimer. Sensorgrams were obtained from an Octet RED96 instrument using a titration series of six concentrations (200–6.25 nM by twofold dilution). **(D)** Neutralization profiles of Ab_d17_-7 and -8 against six tier-2 viruses and two tier-1 viruses, with WT bNAbs PGT121, 124, and 133 included as references. IC_50_ values are highlighted based on the same color-coding scheme as in Figure 4C.

To test this hypothesis, we constructed a new scFv library from the donor-17 PBMCs using IgHV4- and IgLV3-specific primers to target the germline genes of the PGT121 class (Table S1 in Supplementary Material). This library was screened against the trimer probe, with the pre- and post-panning libraries sequenced on the PGM using an Ion 314 v2 chip. NGS yielded 809,354 raw reads, which were analyzed with the H/L-paired *Antibodyomics* pipeline (Table S2C in Supplementary Material). Quantitative library profiles revealed convergence patterns similar to those observed for the diverse donor-17 scFv library (Figure 5A). In brief, IgHV4-59 and IgLV3-21 accounted for over 90% of HCs and LCs upon convergence. The average degree of SHM increased from 12% and 11% to 23% and 27% for HCs and LCs, respectively. In addition, more than 90% of HCs contained long HCDR3 loops of 23–25 aa, characteristic of the PGT121-class bNAbs. Furthermore, the 2D plots demonstrated co-enrichment of PGT124-like HCs and PGT124/PGT133-like LCs, consistent with our findings for the diverse scFv library but with a notable difference in the pattern of distribution (Figure 5B). Nonetheless, the H/L-paired clustering analysis identified three representative scFv clones from the converged library that resembled the WT bNAbs (Figure 6A; Table S3C in Supplementary Material). While the scFv-derived HC database appeared to be enriched for PGT124-like sequences of the IgHV4-59 origin, the PGT133-like LCs were more prevalent than the PGT124-like LCs, showing an ~16-fold difference in their frequencies. Three mAbs (Ab_d17_-7–9) were synthesized for evaluation of antigen binding by ELISA (Figure 6B). Similar to Ab_d17_-1 and Ab_d17_-2 identified from the diverse scFv library, Ab_d17_-7 and Ab_d17_-8 bound to the three N332-containing antigens with EC50 values 10- to 100-fold lower than the WT bNAbs. Recognition of the native-like trimer by Ab_d17_-7 and Ab_d17_-8 was then confirmed by BLI (Figure 6C), which displayed binding profiles comparable to the WT bNAbs (Figure 1C). Consistently, Ab_d17_-7 and Ab_d17_-8 also demonstrated neutralizing breadth and potency almost identical to the PGT121-class bNAbs with some weak activity against a tier-1 HXB2 strain, which could not be neutralized by the WT bNAbs (Figure 6D). Overall, screening of a focused donor-17 scFv library by a native-like trimer probe resulted in mAbs with optimized binding properties and bNAb-like neutralization profiles.

**Figure 7:**
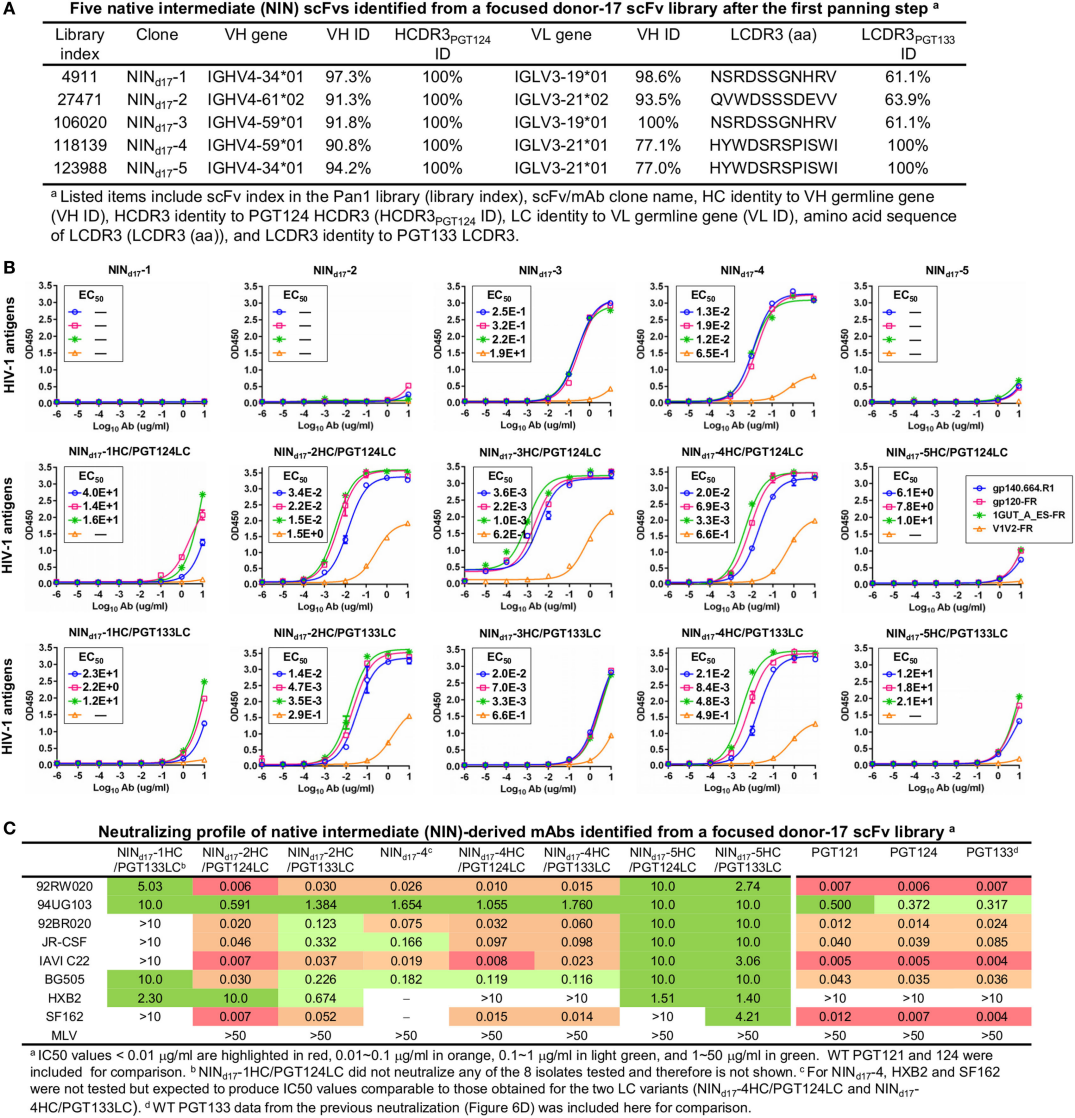
Characterization of the PGT124 intermediates identified from a focused donor-17 single-chain variable fragment (scFv) library. **(A)** Five native intermediates, NIN_d17_-1–5, identified from the Pan1 library based on the HCDR3 analysis. **(B)** Enzyme-linked immunosorbent assay (ELISA) binding of NIN_d17_-1–5 and their LC variants to the gp140.664.R1 trimer and three nanoparticles (gp120-FR, 1GUT_A_ES-FR, and V1V2-FR). The two sets of LC variants were derived by pairing the NIN HCs with PGT124 and PGT133 LCs. EC_50_ values are labeled for all ELISA plots except for instances in which the highest OD_450_ value is below 0.1 or in the cases of ambiguous data fitting. **(C)** Neutralization profiles of selected NIN_d17_ variants against six tier-2 viruses and two tier-1 viruses, with WT broadly neutralizing antibodies PGT121, 124, and 133 included as references. IC_50_ values are highlighted based on the same color-coding scheme as in Figure 4C.

Although trimer panning of two donor-17 scFv libraries converged to the bNAb-like clones with similar functions, visual comparison revealed “islands” of sequences present only on the 2D plots of the focused library, but not the diverse library (Figure 5B). To explore the cause of this discrepancy, we plotted the HC and LC sequences with a CDR3 identity of 95% or greater to the WT bNAbs. Surprisingly, these islands were mainly occupied by sequences with a low level of SHM (<15%) and moderate identity to the WT bNAbs (~80%), suggesting that they may be the native intermediates (termed NINs) of the PGT121-class bNAbs. Interestingly, these NINs were most visible after the first panning step (termed Pan1) but began to disappear as the library was further enriched for high-affinity bNAb-like clones. Five NINs (NIN_d17_-1–5) were selected from the Pan1 library for experimental testing (Figure 7A; Table S4 in Supplementary Material). Of note, sequence analysis revealed that the HCs of all five NINs contained the mature PGT124 HCDR3 loop with a low level of SHM (3–10%) but were assigned to three different germline genes: IgHV4-34, IgHV4-59, and IgHV4-61. While Ab_d17_-2 and NIN_d17_-2 sharing the same IgHV4-61 germline gene with differing levels of SHM suggest an actively evolving PGT124 sub-lineage possessing the 2-aa insertion in HCDR1, the near-germline IgHV4-34 HCs with a mature PGT124 HCDR3 cannot be explained within the current framework of the PGT121-class bNAb lineage development. Due to the random HC/LC pairing in scFv library construction, diverse LCs were observed for the five NINs, with the PGT133-like LCs used by NIN_d17_-4 and -5. Antigen binding of the five NIN-derived mAbs was then assessed by ELISA, with NIN_d17_-3 and -4 displaying notable affinities for the gp140.664.R1 trimer (47), the gp120 nanoparticle (48), and the N332 nanoparticle (Figure 7B) (46). To eliminate the effect of non-functional LCs, we paired the five NIN HCs with the PGT124 and PGT133 LCs. As expected, pairing with the WT bNAb LCs could restore the antigen affinity of NIN_d17_-2, -3, and -4, while moderately increasing the antigen affinity of NIN_d17_-1 and -5. Lastly, a total of nine NIN-derived mAbs were tested for neutralization, with eight showing neutralizing activity (Figure 7C). Overall, NIN mAbs reconstituted from the IgHV4-59 and IgHV4-61 HCs outperformed those reconstituted from the IgHV4-34 HCs, exhibiting neutralization breadth on par with that of the WT bNAbs, although with reduced potency. Furthermore, the IgHV4-59 and IgHV4-61 HCs, when paired with the PGT124 LC, displayed more potent neutralization than their PGT133 LC counterparts. The results also suggested that an SHM level of 10% or lower may be sufficient for the PGT121-class bNAb intermediates to achieve effective Env recognition (by targeting the N332 supersite) and broad HIV-1 neutralization.

**Figure 8:**
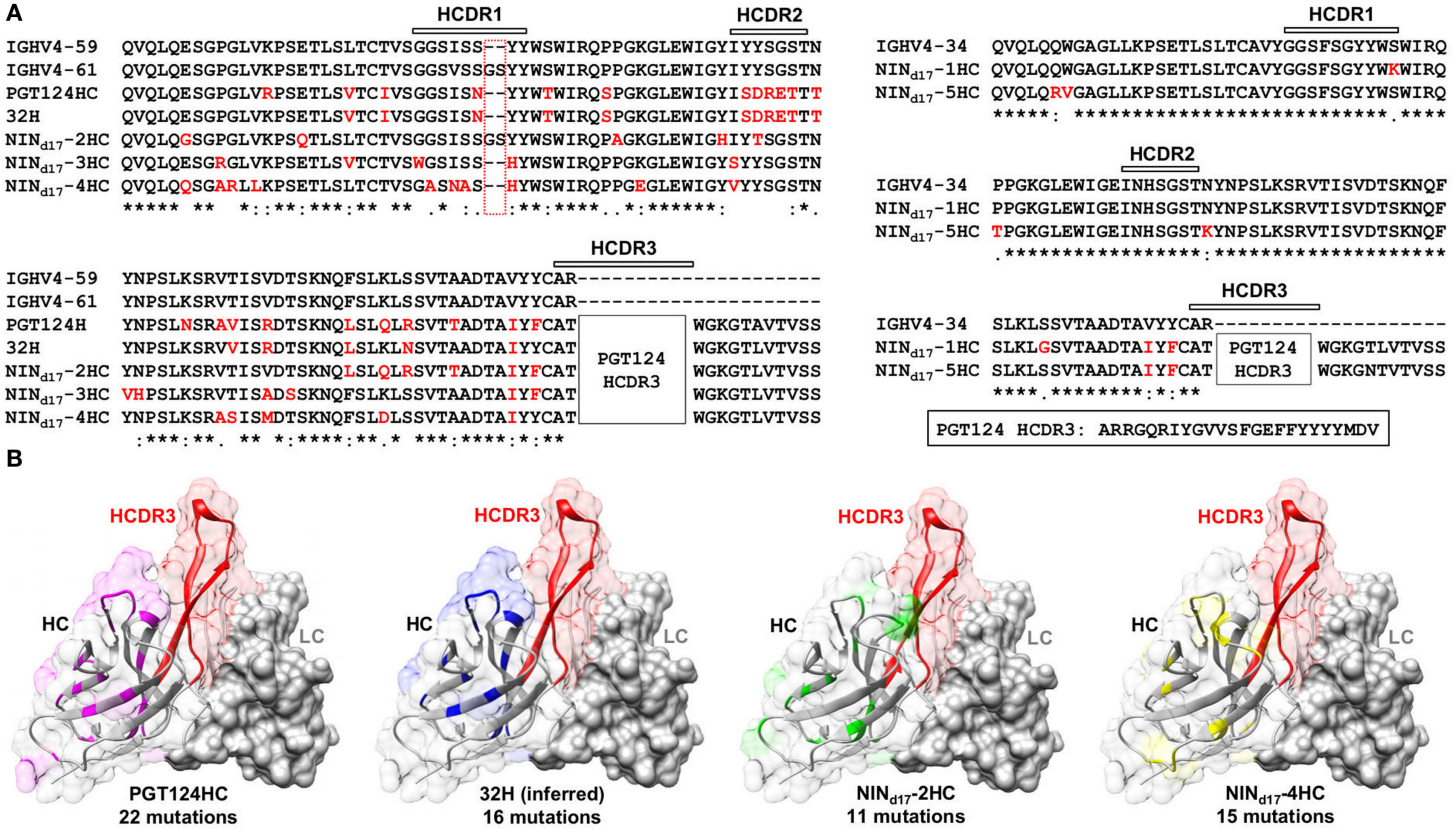
Sequence and structure analyses of the PGT124 intermediates identified from a focused donor-17 single-chain variable fragment (scFv) library. **(A)** Sequence alignment of NIN_d17_-2, -3, and -4 with two HC germline genes (IgHV4-59 and IgHV4-61), PGT124 HC, and inferred 32H (18) (left), and sequence alignment of NIN_d17_-1 and -5 with the germline gene IgHV4-34 (right). The three HCDR regions are marked above the sequences, with the mutations with respect to IgHV4-59 (left) and IgHV3-34 (right) colored in red. As the HCDR3 region in all NIN_d17_ sequences is identical to that of WT PGT124, it is highlighted separately and not shown in the sequence alignment. **(B)** Structural mapping of HC mutations onto the antibody surface for PGT124, inferred intermediate 32H, NIN_d17_-2, and NIN_d17_-4. The structures of antibody variable domains were modeled using the PGT124 structure as a template (PDB ID: 4R2G), with HCs shown as ribbons in transparent molecular surface and LCs as solid gray molecular surface. The HCDR3 loop is colored in red in all models, whereas HC mutations are colored in pink for PGT124, blue for 32H, green for NIN_d17_-2, and yellow for NIN_d17_-4. The number of amino acid mutations with respect to the germline gene (IgHV4-59) is indicated under the structural model.

Sequence alignment and structural modeling revealed distinct features of the newly derived PGT121-class intermediates (Figure 8). For example, compared with the WT PGT124 and an inferred PGT124 intermediate (32H) (18), NIN_d17_-2–4 showed fewer mutations in the HCDR2 loop, suggesting that the highly mutated HCDR2 motif in the mature bNAbs may not be as critical as previously thought. Another important finding was an *N*-linked glycosylation site in the HCDR1 loop of NIN_d17_-4, suggesting that HCDR1 may be a focal point of maturation for facilitating Env interactions through various mechanisms. In contrast, the HCs of NIN_d17_-1 and -5 were IgHV4-34 germline-like sequences with only four to six mutations within the V gene. Furthermore, while the HC mutations within the WT PGT124 and an inferred 32H (18) displayed similar distribution patterns across the protein surface, the HC mutations within NIN_d17_-2 and NIN_d17_-4 were focused mainly on HCDR1 of the functionally important open face (37). Additionally, we identified the NIN-like PGT124 and PGT133 LCs, which contained a similar deletion in FR1 but lacked the 3-aa FR3 insertion compared to the mature bNAbs (Figure S6 in Supplementary Material).

Our results confirmed that PGT124, and a related 10-1074 (36), represents a distinct branch of lineage development with respect to other members of the PGT121 class (18, 40–42), and define the minimal SHM needed to achieve broad HIV-1 neutralization. Since PGT124 and 10-1074 mainly require the N332 glycan for Env recognition (42, 45), and PGT124 intermediates with different levels of SHM have been found in the donor repertoire, the PGT124 sub-lineage may provide a more promising template than PGT121 for immunogen design targeting the N332 supersite.

### PGT124 Intermediates Compete with Trimer-Elicited Mouse Sera for the N332 Supersite

Since the unmutated SOSIP.664 trimer was poorly recognized by the PGT121 precursor, random mutagenesis and structure-based design were undertaken to create sequential trimer immunogens to target the inferred PGT121 precursor and intermediates (18, 31). Although these modified trimers induced bNAb-like responses in Ig knock-in mice (23), they have not been tested in WT animals. In this study, PGT124 intermediates were selected by a native-like trimer probe presenting an intact glycan shield. Based on this finding, we hypothesized that an unmutated trimer may be able to elicit N332-speficic antibody responses in WT animals and that the bNAb intermediates (NINs) identified from the donor-17 scFv library can in turn be used in a competition assay to assess the N332 specificity of serum antibodies elicited by a trimer, or an N332-focused immunogen.

**Figure 9:**
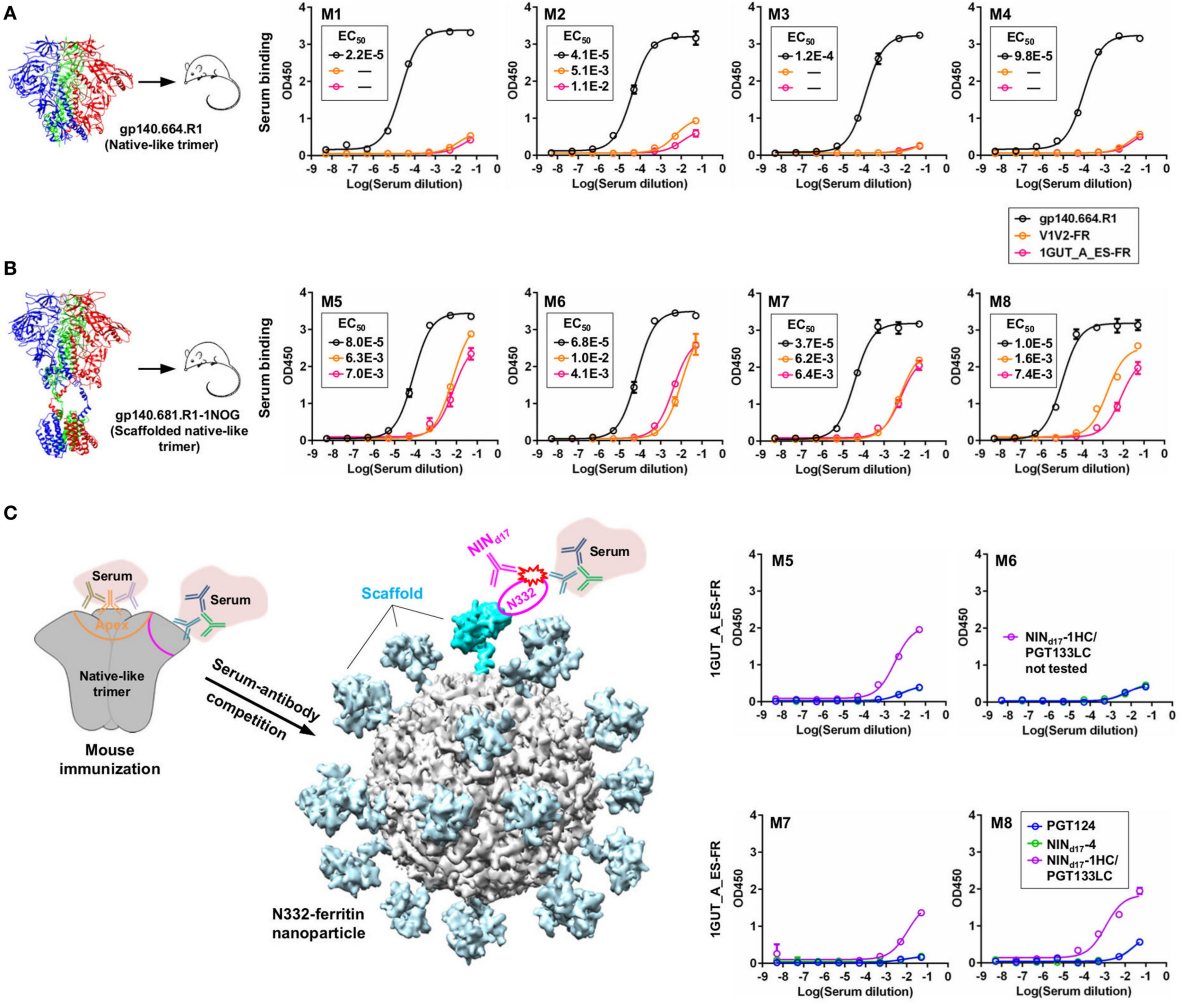
Detection of N332-specific antibody responses in trimer immunization by library-derived PGT124 lineage intermediates. **(A)** Enzyme-linked immunosorbent assay (ELISA) binding of mouse sera elicited by a native-like gp140 trimer (gp140.664.R1) to the trimer immunogen and two nanoparticles (V1V2-FR and 1GUT_A_ES-FR). The structure of this trimer (PDB ID: 5JS9) is shown on the left, with ELISA plots for four subjects in this group (M1–M4) shown on the right. **(B)** ELISA binding of mouse sera elicited by a scaffolded full-length gp140 trimer (gp140.681.R1-1NOG) (39) to the parent trimer (gp140.664.R1) and two nanoparticles (V1V2-FR and 1GUT_A_ES-FR). The structural model of this scaffolded trimer is shown on the left, with ELISA plots for four subjects in this group (M5–M8) shown on the right. **(C)** ELISA binding of mouse sera elicited by the gp140.681.R1-1NOG trimer to an N332 nanoparticle (1GUT_A_ES-FR), in which PGT124 and two intermediate variants (NIN_d17_-4 and NINd17-1HC/PGT133LC) were added to compete for the N332 supersite. A diagram of the competition ELISA is shown on the left, with ELISA plots for four subjects in this group (M5–M8) shown on the right. EC_50_ values are labeled for all ELISA plots except for instances in which the highest OD_450_ value is below 0.1 or in the cases of ambiguous data fitting.

We briefly investigated this hypothesis by examining serum samples from a previous mouse immunization aimed to study the early B-cell responses to the N332 supersite and MPER in the context of multivalent scaffolds and native-like trimers (46). It was reported that a scaffolded full-length gp140 trimer elicited a robust antibody response to the apex, suggesting that the inclusion of MPER and a C-terminal scaffold domain can stabilize the glycan shield and facilitate bNAb recognition (46). In this study, we first performed ELISA to assess sera from mice immunized with the gp140.664.R1 trimer, which was also the basis of the trimer probe used for screening the donor-17 scFv libraries (Figure 9A). Consistent with the previous finding for the SOSIP trimer (102) and with our previous experiment (46), the apex and the N332 supersite were not well recognized by mouse sera, as indicated by two nanoparticle probes. In comparison, enhanced binding to both glycan epitopes was observed for mouse sera elicited by the scaffolded gp140.681.R1-1NOG trimer (Figure 9B), suggesting that an unmutated trimer, when presented in a proper structural context, can induce antibody responses to the bNAb epitopes in the glycan shield. Two library-derived bNAb intermediates, NIN_d17_-1HC/PGT133LC (with weak trimer binding and neutralization) and NIN_d17_-4 (with moderate neutralizing potency), were utilized in a competition ELISA to assess the N332 specificity of mouse sera obtained from the scaffolded trimer group (Figure 9C). The WT PGT124 was included in the ELISA assay as a positive control. As expected, PGT124 and NIN_d17_-4—but not the NIN_d17_-1 variant with a near-germline HC—could effectively block serum binding to the N332 nanoparticle, illustrating the utility of the library-derived bNAb intermediates for immunogen evaluation. It also appeared that murine antibodies, with short (on average 9-aa) HCDR3 loops, failed to compete with human bNAb intermediates with a 24-aa HCDR3 loop poised to penetrate the glycan shield.

## Discussion

Diverse bNAbs identified from the elite neutralizers have served as useful templates for guiding rational HIV-1 vaccine design (103). However, there is a significant disparity between the degrees of SHM observed for bNAbs and for weak- or non-NAb antibody responses in chronic infection and vaccination. The minimal level of SHM required for neutralization and protection is poorly defined, although recent studies of bNAb-virus co-evolution have begun to shed some light on this critical issue (16, 17, 19–22). Limited by patient samples and antibody technologies, it remains a challenge to identify bNAb precursors and intermediates from infected donors, thus hindering B-cell lineage immunogen design (7). To overcome this challenge, germline reversion and phylogenic analysis have been used to derive putative bNAb precursors and intermediates (18). Analyses of the inferred antibodies have revealed distinct patterns for the PGT121 and PGT124 sub-lineages (40–42). These insights constituted the foundation for designing sequential trimer constructs capable of eliciting bNAb-like responses in Ig knock-in mice (23, 31). Despite these encouraging successes, it is necessary to acknowledge the risk in utilizing inferred antibodies resulting from assumptions which may or may not accurately reflect the true events of B-cell lineage development.

A variety of methods has been applied to the identification of HIV-1 bNAbs, including phage display, hybridoma, single B-cell culturing coupled with large-scale functional screening, and antigen-specific single B-cell sorting by flow cytometry (96). Among these, the single-cell methods are considered most advantageous, as they enable isolation of mAbs with natively paired HC and LC from live, functional B cells. It was also suggested that new methods would be necessary for identifying rare precursors and intermediates—a challenge facing both bNAb and immunization studies (96). Advances in NGS technology and templating methods have allowed unbiased analysis of B-cell repertoires during HIV-1 infection and vaccination (15, 17, 46, 101). Presently, NGS is applied to the characterization of scFv antibody libraries but not yet to the direct selection of functional scFv clones due to its insufficient lead length to cover both HC and LC within a scFv (100). In this study, a long-read NGS technology was established that permitted high-throughput sequencing of full-length scFv libraries and used in conjunction with an H/L-paired antibodyomics method for library profiling and clone selection. These advances have transformed the conventional scFv library panning method into a quantitative “digital panning” method and will likely improve the single-cell, H/L-paired antibody isolation and repertoire analysis (104, 105). Using two scFv libraries constructed from the samples of an elite HIV-1 neutralizer (donor-17) (34), we demonstrated the utility of digital panning in the study of PGT121-class bNAbs, with due consideration of recent advances in gp140 trimer design (86, 87). A native-like trimer probe, with structural and antigenic profiles indistinguishable from its parent UFO trimer (47), was utilized to screen donor-17 scFv libraries so as to dissect the details of an N332-dependent bNAb lineage within the antibody repertoire. Digital panning of donor-17 scFv libraries with this trimer probe offered a wealth of novel information on the PGT121 class: PGT122/PGT124 HC variants with a 2-aa HCDR1 insertion, HC and LC intermediates with fewer mutations and different sequence motifs than the inferred intermediates (18), and a versatile open face utilizing HCDR1 and HCDR2 mutations to mediate Env interactions. However, due to the shortcomings of phage display such as primer bias and random H/L pairing, it is possible that only a subset of lineage variants and intermediates were captured. Nonetheless, these new findings will contribute to a more complete understanding of the lineage development of the PGT121-class bNAbs. The bNAb intermediates were also utilized to gauge N332-specific antibody responses elicited by two native-like gp140 trimers. A previously reported gp140 scaffolding strategy (46) appeared to notably enhance the trimer-induced antibody response to the N332 supersite in BALB/c mice, although such response could barely compete with the human bNAb intermediates. Future studies investigating the cause of this enhancement, or evaluating the scaffolded trimers using an extensive regimen, may prove useful for the development of an effective trimer vaccine.

In summary, our study has provided valuable tools, including a native-like trimer probe and the digital panning method, to facilitate bNAb studies, particularly those involving identification of rare bNAb intermediates in HIV-1 patient and vaccination samples. The PGT124 sub-lineage, possessing an invariable HCDR3 loop and multiple library-derived intermediates, may serve as a promising template for B-cell lineage vaccine design targeting the N332 supersite.

## Ethics Statement

Blood samples were acquired from an HIV-1-infected donor (donor-17) of the Protocol G cohort under written consent. The samples were collected following clinical protocols approved by the Republic of Rwanda National Ethics Committee, the Emory University Institutional Review Board, the University of Zambia Research Ethics Committee, the Charing Cross Research Ethics Committee, the UVRI Science and Ethics Committee, the University of New South Wales Research Ethics Committee, the St. Vincent’s Hospital and Eastern Sydney Area Health Service, the Kenyatta National Hospital Ethics and Research Committee, the University of Cape Town Research Ethics Committee, the International Institutional Review Board, the Mahidol University Ethics Committee, the Walter Reed Army Institute of Research (WRAIR) Institutional Review Board, and the Ivory Coast Comité National d’Ethique des Sciences de la Vie et de la Santé (CNESVS).

## Author Contributions

LH and JZ conceived the method. LH, XL, PA, CJM, RA, CDM, and JZ performed digital panning, antibody synthesis, antigen binding assays, and computational analysis. NV, GO, and AW performed negative-stain EM analysis of the gp140 trimer probe. BZ set up the MicroPulser system. KS-F, DS, and DB performed the HIV-1 neutralization assays. LH and JZ wrote the manuscript with input from all coauthors.

## Conflict of Interest Statement

The authors declare that the research was conducted in the absence of any commercial or financial relationships that could be construed as a potential conflict of interest.

## Supplementary Material

Figure S1 Negative-stain EM of the biotinylated, Avi-tagged BG505 gp140 trimer probe used for biopanning. This trimer probe contains a redesigned heptad repeat 1 bend (47) and a biotinylated Avi-tag located immediately downstream of residue 664 (termed gp140.664.R1-Avi-Biot). **(A)** Raw micrograph of the BG505 gp140.664.R1-Avi-Biot trimer. **(B)** Reference-free 2D class averages of the BG505 gp140.664.R1-Avi-Biot trimer. Percentages of native-like trimers (closed and partially open or breathing trimers) and non-native species (misfolded trimers as well as dimers and monomers) are indicated. **(C)** The estimated resolution (~21 Å) of the EM reconstruction was calculated from the Fourier Shell Correlation (FSC) using a cutoff of 0.5. **(D)** Top and side views of the 3D EM reconstruction of the BG505 gp140 SOSIP.664 trimer and the gp140.664.R1-Avi-Biot trimer. The EM densities of the SOSIP trimer are shown in gray transparent surface with the crystal structure (PDB 4TVP, gp120 in blue with V1V2 in magenta, V3 in green and gp41 in brown) fitted into the density. The EM densities of the gp140.664.R1-Avi-Biot trimer are shown in gray transparent surface with the SOSIP trimer densities overlaid as wire mesh (in orange). The contour level used for the gp140.664.R1-Avi-Biot trimer density was ~33.

Figure S2 Assessment of the *Antibodyomics* 2.0 pipeline. **(A)** Schematic view of the chain-specific *Antibodyomics* pipeline, which consists of five steps including (1) data reformatting and cleaning, (2) germline gene assignment, (3) sequencing error correction, (4) calculation of sequence identity to a set of known antibodies, and (5) determination of CDR3 and variable domain boundaries. Step 3 (error correction) is highlighted with a red dashed-line box. **(B)** Distribution of improvement in sequence quality resulting from error correction. Quality improvement is measured by the change of amino acid sequence identity with respect to the germline V gene. For the donor-17 antibody chain data generated by 454 sequencing (18), *Antibodyomics* 1.0 yielded an average improvement of 19.4 and 21.7% for HC and LC, respectively, in comparison with 22.5 and 24.1% from *Antibodyomics* 2.0.

Figure S3 Ultra-deep sequencing of the donor-17 HC repertoire. **(A)** Distributions of germline gene usage (left), somatic hypermutation (middle), and HCDR3 length (right). In the histogram of germline gene family distribution, IgHV4 is highlighted in black whereas other germline genes are shown in gray (left 1). A more detailed distribution within the IgHV4 family is also plotted (left 2). **(B)** Identity-divergence analysis of the donor-17 HC repertoire using the PGT121-class bNAb HCs as templates. Sequences are plotted as a function of sequence identity to WT bNAb HCs and germline divergence. Color-coding indicates sequence density at a particular point on the 2D plot. Wild-type bNAb HCs are labeled on the 2D plots as black dots. Sequences with HCDR3 identity of 90% or greater and those assigned to IgHV4-61 are shown as orange dots and red asterisks, respectively, with the number of sequences for each labeled on the 2D plot. **(C)** Sequence alignment of selected HCs of the putative IgHV4-61 origin with respect to two germline genes (IgHV4-59 and IgHV4-61) and WT bNAb HCs. The three HCDR regions are marked above the sequences, with the mutations with respect to IgHV4-59 colored in red. Below the sequence alignment, asterisk (*) indicates identical residues, colon (:) indicates residues with strongly similar properties, and period (.) indicates residues with weakly similar properties.

Figure S4 Digital panning of a diverse donor-17 single-chain variable fragment (scFv) library against a clade-C V1V2-ferritin nanoparticle. This scFv library, constructed from the donor-17 peripheral blood mononuclear cells (PBMCs) using a large set of primers, has been screened against a native-like gp140 trimer probe, gp140.664.R1-Avi-Biot. Distributions of germline gene usage **(A)**, somatic hypermutation **(B)**, and CDR3 loop length **(C)** are plotted for the five scFv libraries obtained from the nanoparticle panning process. Histograms are color-coded according to their antigen panning steps: gray (Pan0), cyan (Pan1), green (Pan2), orange (Pan3), and red (Pan4).

Figure S5 Identification and characterization of monoclonal antibodies (mAbs) from a diverse donor-17 single-chain variable fragment (scFv) library screened against a clade-C V1V2-ferritin nanoparticle. **(A)** Six prevalent scFv clones identified by H/L-paired, CDR3-based clustering analysis. **(B)** Enzyme-linked immunosorbent assay (ELISA) binding of three representative bNAbs of the PGT121 class (PGT121, 124, and 133) and six scFv-derived mAbs (VAb_d17_-1–6) to four HIV-1 antigens including a native-like trimer (gp140.664.R1), a gp120-ferritin nanoparticle (gp120-FR), an N332 nanoparticle (1GUT_A_ES-FR), and a V1V2-ferritin nanoparticle (V1V2-FR). For VAb_d17_s, ferritin was included in the ELISA as a negative control. EC_50_ values are labeled for all ELISA plots except for instances in which the highest OD_450_ value is below 0.1 or in the cases of ambiguous data fitting.

Figure S6 Additional native intermediates (NINs) selected from a focused donor-17 single-chain variable fragment (scFv) library during the trimer panning process. **(A)** Sequence alignment of HCs with assigned germline genes and WT PGT124 HC. **(B)** Sequence alignment of LCs with germline gene IgLV3-21 and WT PGT124 LC or PGT133 LC. The three HCDR regions are marked above the sequences, with the mutations with respect to the assigned germline gene colored in red. Below the sequence alignment, asterisk (*) indicates identical residues, colon (:) indicates residues with strongly similar properties, and period (.) indicates residues with weakly similar properties.
