# Supplementary material for: Hidden Lineage Complexity of Glycan-Dependent HIV-1 Broadly Neutralizing Antibodies Uncovered by Digital Panning and Native-Like gp140 Trimer
Source: Front Immunol. 2017 Aug 24;8:1025. doi: 10.3389/fimmu.2017.01025 (PMC5573810; doi:10.3389/fimmu.2017.01025)
Supplement: Figure S1 — Negative-stain EM of the biotinylated, Avi-tagged BG505 gp140 trimer probe used for biopanning. This trimer probe contains a redesigned heptad repeat 1 bend (47) and a biotinylated Avi-tag located immediately downstream of residue 664 (termed gp140.664.R1-Avi-Biot). (A) Raw micrograph of the BG505 gp140.664.R1-Avi-Biot trimer. (B) Reference-free 2D class averages of the BG505 gp140.664.R1-Avi-Biot trimer. Percentages of native-like trimers (closed and partially open or breathing trimers) and non-native species (misfolded trimers as well as dimers and monomers) are indicated. (C) The estimated resolution (~21 Å) of the EM reconstruction was calculated from the Fourier Shell Correlation (FSC) using a cutoff of 0.5. (D) Top and side views of the 3D EM reconstruction of the BG505 gp140 SOSIP.664 trimer and the gp140.664.R1-Avi-Biot trimer. The EM densities of the SOSIP trimer are shown in gray transparent surface with the crystal structure (PDB 4TVP, gp120 in blue with V1V2 in magenta, V3 in green and gp41 in brown) fitted into the density. The EM densities of the gp140.664.R1-Avi-Biot trimer are shown in gray transparent surface with the SOSIP trimer densities overlaid as wire mesh (in orange). The contour level used for the gp140.664.R1-Avi-Biot trimer density was ~33. [file Presentation_1.PDF]

TABLE S1| Primers used for the construction of single-chain variable fragment (scFv) libraries from the donor-17 samples. [a]

**H-chain 5' primers**	
HuVH1B/7A-Sfil-F	GCGGCCCAGCCGGCCATGGCA CAG RTG CAG CTG GTG CAR TCT GG
HuVH1C-Sfil-F	GCGGCCCAGCCGGCCATGGCA SAG GTC CAG CTG GTR CAG TCT GG
HuVH2B-Sfil-F	GCGGCCCAGCCGGCCATGGCA CAG RTC ACC TTG AAG GAG TCT GG
HuVH3B-Sfil-F	GCGGCCCAGCCGGCCATGGCA SAG GTG CAG CTG GTG GAG TCT GG
HuVH3C-Sfil-F	GCGGCCCAGCCGGCCATGGCA GAG GTG CAG CTG GTG GAG WCY GG
HuVH4B-SfiI-F	GCGGCCCAGCCGGCCATGGCA CAG GTG CAG CTA CAG CAG TGG GG
HuVH4C- SfiI-F	GCGGCCCAGCCGGCCATGGCA CAG STG CAG CTG CAG GAG TCS GG
HuVH5B-SfiI-F	GCGGCCCAGCCGGCCATGGCA GAR GTG CAG CTG GTG CAG TCT GG
HuVH6A-SfiI-F	GCGGCCCAGCCGGCCATGGCA CAG GTA CAG CTG CAG CAG TCA GG
**H-chain 3' primers**	
HuJH1/2-linker-R	AGAGCCACCTCCGCCTGAACCGCCTCCACC TGA GGA GAC GGT GAC CAG GGT GCC
HuJH3-linker-R	AGAGCCACCTCCGCCTGAACCGCCTCCACC TGA AGA GAC GGT GAC CAT TGT CCC
HuJH4/5-linker-R	AGAGCCACCTCCGCCTGAACCGCCTCCACC TGA GGA GAC GGT GAC CAG GGT TCC
HuJH6-linker-R	AGAGCCACCTCCGCCTGAACCGCCTCCACC TGA GGA GAC GGT GAC CGT GGT CCC
**κ-chain 5' primers**	
HuVK1B-linker-F	GGCGGAGGTGGCTCTGGCGGTGGCGGATCG GAC ATC CAG WTG ACC CAG TCT CC
HuVK2-linker-F	GGCGGAGGTGGCTCTGGCGGTGGCGGATCG GAT GTT GTG ATG ACT CAG TCT CC
HuVK3B-linker-F	GGCGGAGGTGGCTCTGGCGGTGGCGGATCG GAA ATT GTG WTG ACR CAG TCT CC
HuVK4B-linker-F	GGCGGAGGTGGCTCTGGCGGTGGCGGATCG GAT ATT GTG ATG ACC CAC ACT CC
HuVK5-linker-F	GGCGGAGGTGGCTCTGGCGGTGGCGGATCG GAA ACG ACA CTC ACG CAG TCT CC
HuVK6-linker-F	GGCGGAGGTGGCTCTGGCGGTGGCGGATCG GAA ATT GTG CTG ACT CAG TCT CC
**κ-chain 3' primers**	
HSCJK14oB-SfiI-R	TTGGCCTCCCGGGCCACTAGT TTT GAT YTC CAC CTT GGT CCC
HSCJK2oB-SfiI-R	TTGGCCTCCCGGGCCACTAGT TTT GAT CTC CAG CTT GGT CCC
HSCJK3oB-SfiI-R	TTGGCCTCCCGGGCCACTAGT TTT GAT ATC CAC TTT GGT CCC
HSCJK5oB-SfiI-R	TTGGCCTCCCGGGCCACTAGT TTT AAT CTC CAG TCG TGT CCC
**λ-chain 5' primers**	
HuVL1A-linker-F	GGCGGAGGTGGCTCTGGCGGTGGCGGATCG CAG TCT GTG CTG ACT CAG CCA CC
HuVL1B-linker-F	GGCGGAGGTGGCTCTGGCGGTGGCGGATCG CAG TCT GTG YTG ACG CAG CCG CC
HuVL1C-linker-F	GGCGGAGGTGGCTCTGGCGGTGGCGGATCG CAG TCT GTC GTG ACG CAG CCG CC
HuVL2-linker-F	GGCGGAGGTGGCTCTGGCGGTGGCGGATCG CAR TCT GCC CTG ACT CAG CCT
HuVL3A-linker-F	GGCGGAGGTGGCTCTGGCGGTGGCGGATCG TCC TAT GWG CTG ACT CAG CCA CC
HuVL3B-linker-F	GGCGGAGGTGGCTCTGGCGGTGGCGGATCG TCT TCT GAG CTG ACT CAG GAC CC
HuVL4-linker-F	GGCGGAGGTGGCTCTGGCGGTGGCGGATCG CAC GTT ATA CTG ACT CAA CCG CC
HuVL5-linker-F	GGCGGAGGTGGCTCTGGCGGTGGCGGATCG CAG GCT GTG CTG ACT CAG CCG TC
HuVL6-linker-F	GGCGGAGGTGGCTCTGGCGGTGGCGGATCG AAT TTT ATG CTG ACT CAG CCC CA
HuVL7/8-linker-F	GGCGGAGGTGGCTCTGGCGGTGGCGGATCG CAG RCT GTG GTG ACY CAG GAG CC
HuVL9-linker-F	GGCGGAGGTGGCTCTGGCGGTGGCGGATCG CWG CCT GTG CTG ACT CAG CCM CC
PGT121L-linker-F[b]	GGCGGAGGTGGCTCTGGCGGTGGCGGATCG TATGTG TCCGATATATCTGTGGCCCCAGG
PGT122L-linker-F[b]	GGCGGAGGTGGCTCTGGCGGTGGCGGATCG TATGTG ACCTTTGTGTCAGTGGCCCCAGG
PGT123L-linker-F[b]	GGCGGAGGTGGCTCTGGCGGTGGCGGATCG TATGTG TCCTCTATGTCCGTGTCCCCGGG
PGT124L-linker-F[b]	GGCGGAGGTGGCTCTGGCGGTGGCGGATCG TCCTATGTG AGCCCACTGTCAGTGGCCCT
PGT133L-linker-F[b]	GGCGGAGGTGGCTCTGGCGGTGGCGGATCG TCGTTAAACCCACTGTCGCTGGCCCCAGG
**λ-chain 3' primers**	
HSCJL1236-SfiI-R	TTGGCCTCCCGGGCCACTAGT TAG GAC GGT CAS CTT GGT SCC
HSCJL4-SFiI-R	TTGGCCTCCCGGGCCACTAGT TAA AAT GAT CAG CTG GGT TCC
HSCJL57-SfiI-R	TTGGCCTCCCGGGCCACTAGT GAG GAC GGT CAG CTS GGT SCC
**scFv PCR primers** [c]	
sfiI-F	GCAGCCGCTGGATTGTTATTACTCGCGGCCCAGCCGGCCATGGCA
sfiI-R	TTTGTCATCGTCATCTTTGTAGTCTTGGCCTCCCGGGCCACTAGT
**NGS primers**	
fP1-SfiI-F	CCACTACGCCTCCGCTTTCCTCTCTATGGGCAGTCGGTGAT GCGGCCCAGCCGGCCATGGCA
A-SfiI-[Barcode]-R	CCATCTCATCCCTGCGTGTCTCCGACTCAG [Barcode] TTGGCCTCCCGGGCCACTAG

[a] The primers were compiled from those listed in Zhu et al., *Methods Mol Biol* 2009, 525:129 (Ref 54) and the book "Phage Display – A Laboratory Manual" Chapter 9 (pg.924).

[b] Due to a large deletion in the framework region 1 (FR1), PGT121 family-specific λ-chain primers were designed to capture PGT121-like light chains.

[c] fP1 and A, highlighted in the cyan shade, are PGM sequencing adaptors. In this study, five Ion Xpress[TM] barcodes (in brackets) were used to differentiate the five donor-17 scFv libraries, Pan0-Pan4, pooled in one NGS run.

TABLE S2| Long-read deep sequencing and antibodyomics pipeline processing of donor-17 scFv libraries. [a]

N_{tot}	Library ID	$N_{seq\text{-}lib}$	<Length> (nt)	N_{assign}	Chain	N_{chain}	N_{chain}-P	$Perc_{use}$
A. Digital panning of a diverse donor-17 scFv library against the native-like trimer probe.								
					H	61,790	58,676	20.8%
	Pan0	320,421	777	282,413	κ	47,851	13,793	4.9%
					λ	164,255	44,883	15.9%
					H	29,337	27,998	19.7%
	Pan1	159,506	781	142,466	κ	25,472	7,008	4.9%
					λ	81,051	20,990	14.8%
					H	10,951	10,590	8.5%
1,029,451	Pan2	131,656	814	125,026	κ	2,426	550	0.4%
					λ	93,224	10,040	8.1%
					H	10,191	9,840	8.4%
	Pan3	119,848	811	116,907	κ	608	132	0.1%
					λ	91,911	9,708	8.3%
					H	8,987	8,679	7.3%
	Pan4	123,712	817	119,232	κ	426	76	0.1%
					λ	90,644	8,603	7.2%
B. Digital panning of a diverse donor-17 scFv library against a clade-C V1V2-ferritin nanoparticle (negative control).								
					H	28,145	26,448	16.5%
	Pan0	171,308	771	160,121	κ	27,673	6,599	4.1%
					λ	94,344	19,849	12.4%
					H	15,888	15,025	15.1%
	Pan1	107,826	767	99,391	κ	20,335	4,689	4.7%
					λ	54,041	10,336	10.4%
					H	16,068	15,302	16.2%
693,016	Pan2	99,810	775	94,276	κ	22,204	5,465	5.8%
					λ	51,365	9,837	10.4%
					H	11,609	11,184	14.0%
	Pan3	83,120	767	79,899	κ	7,087	2,549	3.2%
					λ	57,929	8,635	10.8%
					H	22,499	22,101	21.0%
	Pan4	109,005	778	105,092	κ	1,092	393	0.4%
					λ	91,342	21,708	20.6%
C. Digital panning of a focused donor-17 scFv library against the native-like trimer probe.								
	Pan0	258,714	791	236,581	H	70,911	67,594	28.6%
					λ	184,128	67,594	28.6%
	Pan1	136,899	795	126,054	H	35,706	34,361	27.3%
					λ	99,693	34,361	27.3%
809,354	Pan2	123,287	816	117,564	H	35,526	33,815	28.8%
					λ	93,859	33,815	28.8%
	Pan3	90,152	807	88,121	H	29,896	28,951	32.9%
					λ	75,073	28,951	32.9%
	Pan4	106,073	816	101,910	H	30,095	28,788	28.2%
					λ	82,466	28,788	28.2%

[a] Listed items include the total number of reads, panning library ID, number of scFv sequences within each library, average read length of the scFv sequences, number of scFv sequences that can be reliably assigned to a LC germline gene with a cutoff E-value of 10^{-3}, antibody chain type (H, κ, and λ), number of full-length HCs and LCs after the *Antibodyomics* pipeline processing, number of paired HC-LC sequences, and percentage of HCs and LCs used for frequency analysis and clone selection (calculated as $N_{chain}\text{-}P/N_{assign}\times100$).

TABLE S3| Amino acid sequences of mAbs selected from donor-17 scFv libraries after biopanning against HIV-1 antigens. [a]

scFv/mAb	HC name	HC sequence	LC name	LC sequence
A. Representative mAb sequences selected from a diverse donor-17 scFv library after biopanning against the native-like trimer probe				
Ab_{d17}-1	T2P4H1	QVQLQESGPGLVKPSETLSLTCTVSGGSISNYYWTWIRQSPGKGLEWIGYISDRETTTYNPSLNSRAVISRDTSKNQLSLQLRSVTTADTAIYFCATARRGQRIYGVVSFGEFFYYYYMDVWGKGTTVTVSS	T1P4L1	SLNPLSVAPGATAKIPCGERSRGSRAVQWYQQKPGQAPTLIIYNNQDRPAGVSERFSGNPDVAIGVTATLTISRVEVGDEADYYCHYWDSRSPISWIFGGGTKLTVL
Ab_{d17}-2	T2P4H2	QLQLQESGPGLVKPSQTLSLTCTVSGGSISSGGYYWTWIRQSPGKGLEWIGYISDRETTTYNPSLNSRAVISRDTSKNQLSLQLRSVTTADTAIYFCATARRGQRIYGVVSFGEFFYYYYMDVWGKGTTVTVSS	T1P4L2	SYVSPLSVALGETARISCGRQALGSRAVQWYQHKPGQAPILLIYNNQDRPSGIPERFSGTPDINFGTTATLTISGVEVGDEADYYCHMWDSRSGFSWSFGGGTKLTVL
Ab_{d17}-3	T2P4H1[b]	See above	T2P4L3	QSVLTQPPSVSGAPGQRVTISCTGSTSNIGAGYDVHWYQQLPGTAPKLLIYANNNRPSGVPDRFSGSKSGASASLAITGLQAEDEAEYYCQSYDSSLSGVVFGGGTQLTVL
Ab_{d17}-4	T2P4H3	QVQLVASGGGVVQPGGSLRLSCAASGFNFKNYAMHWVRQAPGRGPEWVAIIWFDGSKEYYADSVKGRFSLSRDNSENTLYLQMDSLRVDDTAIYYCVSDGGDPDTPWFIGAFDVWGQGTMVTVSS	T2P4L3[b]	See above
Ab_{d17}-5	T2P4H4	EVQLVQSGTEVKKPGASVRVSCKASGYSFTDYGMNWVRQAPGQGLEWMGWITTDTGDPTYAQGFTGRFVFSLDTSVSTAYLEIYSLKPEDTAVYYCARATAPWSHWGQGTLVTVSS	T2P4L4	QAVVTQEPSLTVSPGGTVTLTCGSSTGAVTTGFYPNWFQQKPGQAPKSLIYSTSNKYSWTPARFSGSLLGDKAALTVSGVQPEDEAEYYCLLYHSGAQPYVVFGGGTQLTVL
Ab_{d17}-6	T2P4H5	QVQLVQSGAEVKKPGASVRVACKASGYTFRHHGVSWVRQAPGQGLEWMGWISAYNGNTNYAQKVQGRVTMTTDSSTNTAYMELRSLRSDDTAIYYCARDGEWQIMNYYYKGMDVWGQGTTVTVSS	T2P4K5	DIQLTQSPSTLSASVGDKVTITCRASQSISSWLAWYQQKPGKAPKLLIYKASNLESGVPFRFSGSGSGTEFTLTVSSLQPEDFATYYCQQSFTTPQTFGQGTKLEIK
B. Representative mAb sequences selected from a diverse donor-17 scFv library after biopanning against a clade-C V1V2-ferritin nanoparticle (negative control)				
VAb_{d17}-1	V1P4H1	QVQLVESGAEVKKPGASVKVSCKASGYTFTRYYMHWVRQAPGQGLEWMGRINPNSGGTNYAQKFQGRVTMTRDTSISTAYMELSRLRSDDTAVYYCATGWGLRGQPDGYWGQGTLVTVSS	V1P4L1	QTVVTQEPSLTVSPGGTVTLTCGSSTGAVTSGHYPYWFQQKPGQAPRTLIYGASNKHSWTPARFSGSLLGVKAALTLSGAQPEDEADYYCLLSFGGAWVFGGGTQLTVL
VAb_{d17}-2	V1P4H2	EVQLVESGGQLVQPGGSLRLSCAASGFTFSSYAMSWVRQAPGKGLEWVSLISASGASTYYADSVKGRFTISRDNSKNTLYLQMNSLRAEDTAVYFCAKGGLLPPHFDYWGQGTLVTVSS	V1P4L1[b]	See above
VAb_{d17}-3	V1P4H2[b]	See above	V1P4L2	NFMLTQPHSVSESPGKTVTISCTRSSGNIASNFVQWYQQRPGSSPTTVIYEDNQRPSGVPDRFSGSIDGSSNSASLTISGLQTEDEADYYCQTYDRSNVVFGGGTQLTVL
VAb_{d17}-4	V1P4H3	QVQLVQSGAEVKKPGASVKVSCKASGYTFNNWYMHWVRQAPGQGLEWMGVIDPSGGMRTIYAQKLQGRVTMTRDTSTSTIYMELNNLTSEDTAVYYCARAHYDSSTWGGFAYWGQGTLVTVSS	V1P4L1[b]	See above
VAb_{d17}-5	V1P4H4	QVQLVQSGAEVKKPGASVKVSCKASGYTFTGYYMHWVRQAPGQGLEWMGWINPNSGGTNYAQKFQGRVTMTRDTSTSTVYMEMSSLRSEDTAVYYCAREEYRGDHDAFDIWGQGTLVTVSS	V1P4K3	DIQLTQSPSSLSASVGDRVTITCRASQGIRNDLGWYQQKPGKAPKLLIYAASSLQSGVPSRFSGSGSGTDFTLTISSLQPEDFATYYCLQDYNYPYTFGQGTKLEIK
VAb_{d17}-6	V1P4H5	EVQLVQSGAEVKKPGESLKISCKGSGYSFTSYWIGWVRQMPGKGLEWMGIIYPGDSDTRYSPSFQGQVTISADKSISTAYLQWSSLKASDTAMYYCASLYGSGSYYDYWGQGTLVTVSS	V1P4L1[b]	See above
C. Representative mAb sequences selected from a focused donor-17 scFv library after biopanning against the native-like trimer probe				
Ab_{d17}-7	T1P4H1	QLQLQESGPGLVKPSETLSVTCIVSGGSISNYYWTWIRQSPGKGLEWIGYISDRETTTYNPSLNSRAVISRDTSKNQLSLQLRSVTTADTAIYFCATARRGQRIYGVVSFGEFFYYYYMDVWGKGTTVTVSS	T1P4L1[b]	
Ab_{d17}-8	T1P4H1[b]	See above	T1P4L2[b]	
Ab_{d17}-9	T1P4H1[b]	See above	T1P4L3	SSELTQDPAVSVALGQTVRITCQGDSLRSYYASWYQQKPGQAPVLVIYGKNNRPSGIPDRFSGSSSGNTASLTITGAQAEDEADYYCNSRDSSGNHLVFGGGTKLTVL

[a] Listed items include scFv/mAb name, HC name, HC sequence, LC name, and LC sequence.
[b] The same HC or LC may be used by multiple scFv clones due to random HC/LC pairing in scFv library construction. In such case, the sequence is only shown once.

TABLE S4| Amino acid sequences of native intermediates (NINs) selected from a focused donor-17 scFv library after trimer panning. [a]

Lib index	scFv/mAb	HC sequence	LC sequence
4911	NIN_{d17}-1	QVQLQQWGAGLLKPSETLSLTCAVYGGSFSGYYWKWIRQPPGKGLEWIGEINHSGSTNYN PSLKSRVTISVDTSKNQFSLKLGSVTAADTAIYFCATARRGQRIYGVVSFGEFFYYYYMD VWGKGTLVTVSS	SSELTQDPAVSVTLGQTVRITCQGDSLRSFYASWYQQKPEQAPVLVIYGKNNRPSGIPDR FSGSSSGNTASLTITGAQAEDEADYYCNSRDSSGNHRVFGGSTKLTVL
27471	NIN_{d17}-2	QVQLQGSGPGLVKPSQTLSLTCTVSGGSISSGSYYWSWIRQPAGKGLEWIGHIYTSGSTN YNPSLKSRVTISVDTSKNQLSLQLRSVTTADTAIYFCATARRGQRIYGVVSFGEFFYYYY MDVWGKGTLVTVSS	SYVTFVSVAPGQTATVTCGGNNIGSKSVHWFQQKPGQAPVLVLYDDDDRPSGIPEQFSGS KSGSTASLTISRVEAGDEADYYCQVWDSSSDEVVFGGGTKLTVL
106020	NIN_{d17}-3	QVQLQESGRGLVKPSETLSVTCTVSWGSISSHYWSWIRQPPGKGLEWIGYSYYSGSTNVH PSLKSRVTISADSSKNQFSLKLSSVTAADTAIYFCATARRGQRIYGVVSFGEFFYYYYMD VWGKGTLVTVSS	SSELTQDPAVSVALGQTVRITCQGDSLRSYYASWYQQKPGQAPVLVIYGKNNRPSGIPDR FSGSSSGNTASLTITGAQAEDEADYYCNSRDSSGNHRVFGGGTQLTVL
118139	NIN_{d17}-4	QVQLQQSGARLLKPSETLSLTCTVSGASNASHYWSWIRQPPGEGLEWIGYVYYSGSTNYN PSLKSRASISMDTSKNQFSLDLSSVTAADTAIYYCATARRGQRIYGVVSFGEFFYYYYMD VWGKGTLVTVSS	SSYVSPLSVAPGATAKIPCGERSRGSRAVQWYQQKPGQAPTLIIYNNQDRPAGVSERFSG NPDVAIGVTATLTISRVEVGDEADYYCHYWDSRSPISWIFGGGTKVTVL
123988	NIN_{d17}-5	QVQLQRVGAGLLKPSETLSLTCAVYGGSFSGYYWSWIRQTPGKGLEWIGEINHSGSTKYN PSLKSRVTISVDTSKNQFSLKLSSVTAADTAIYFCATARQGQRIYGVVSFGEFFYYYYMD VWGKGNTVTVSS	SYVTFVSVAPGATAKIPCGERSRGSRAVQWYQQKPGQAPTLIIYNNQDRPAGVSERFSGN PDVAIGVTATLTISRVEVGDEADYYCHYWDSRSPISWIFGGGTKLTVL

[a] Listed items include library index, scFv/mAb name, HC sequence, and LC sequence.

FIGURE S1

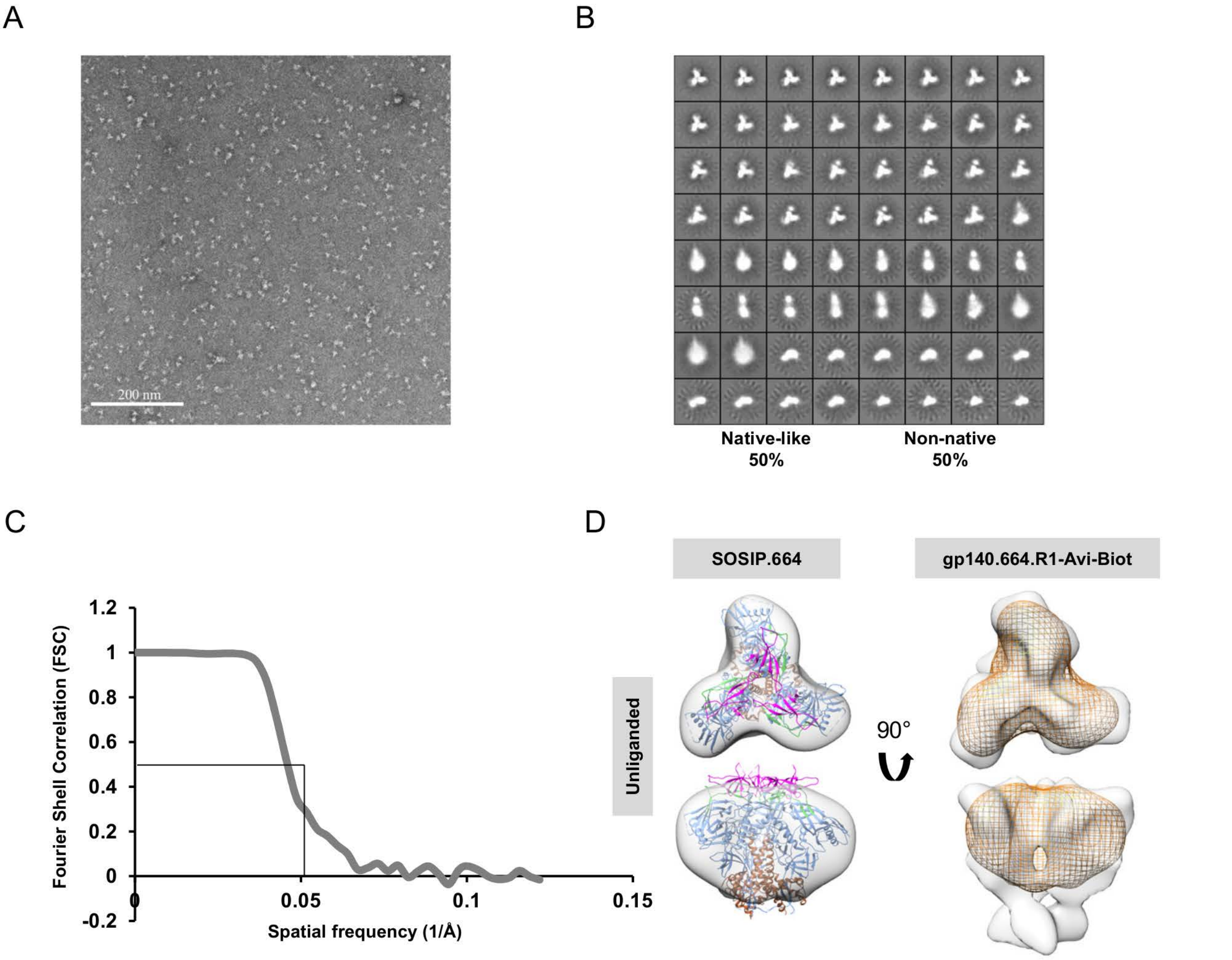
A
200 nm
B
Native-like
50%
Non-native
50%
C
Fourier Shell Correlation (FSC)
Spatial frequency (1/Å)
1.2
1
0.8
0.6
0.4
0.2
0
-0.2
0
0.05
0.1
0.15
D
SOSIP.664
gp140.664.R1-Avi-Biot
Unliganded
90°

FIGURE S2

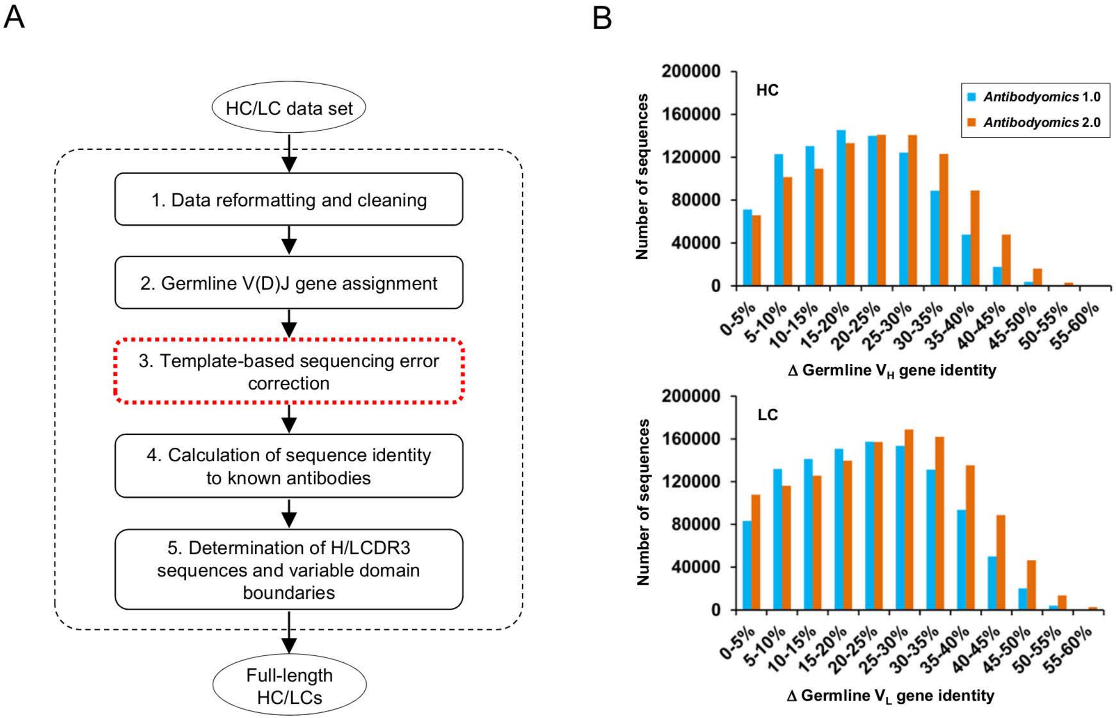
A
HC/LC data set
1. Data reformatting and cleaning
2. Germline V(D)J gene assignment
3. Template-based sequencing error correction
4. Calculation of sequence identity to known antibodies
5. Determination of H/LCDR3 sequences and variable domain boundaries
Full-length HC/LCs
B
HC
Antibodyomics 1.0
Antibodyomics 2.0
Number of sequences
200000
160000
120000
80000
40000
0
0-5%
5-10%
10-15%
15-20%
20-25%
25-30%
30-35%
35-40%
40-45%
45-50%
50-55%
55-60%
Δ Germline V_H gene identity
LC
Number of sequences
Δ Germline V_L gene identity

FIGURE S3

A

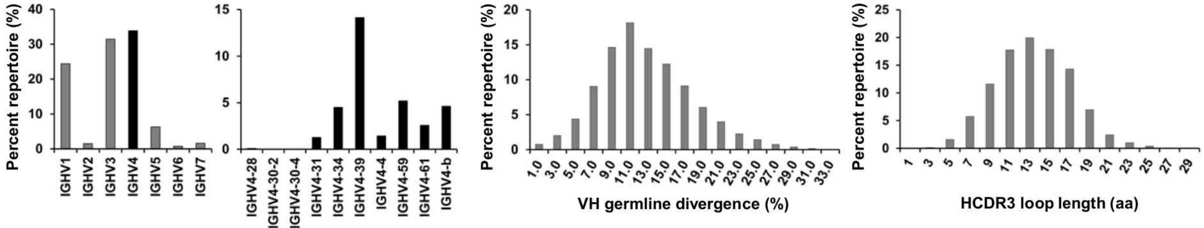

B

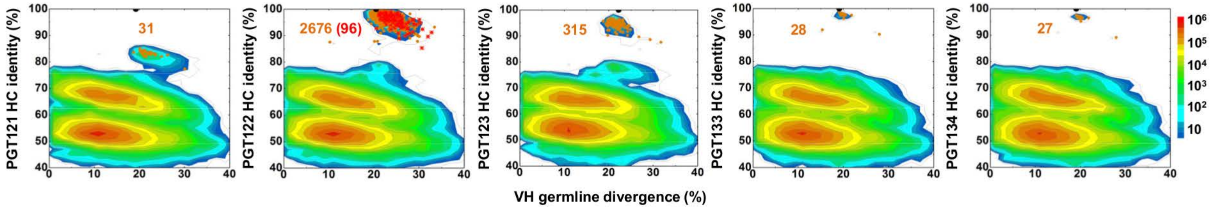

C

```
                                   HCDR1                          HCDR2
IGHV4-59     QVQLQESGPGLVKPSETLSLTCTVSGGSISS--YYWSWIRQPPGKGLEWIGYIYYSGSTNYNPSLKS
IGHV4-61     QVQLQESGPGLVKPSETLSLTCTVSGGSVSSGSYYWSWIRQPPGKGLEWIGYIYYSGSTNYNPSLKS
PGT122HC     QVHLQESGPGLVKPSETLSLTCNVSGTLVRD--NYWSWIRQPLGKQPEWIGYVHDSGDTNYNPSLKS
14297408     TVHLQESGPGLVKPSETLSLTCNVSGTSVRRDNYYWSWIRQPLGKQPEWIGYVHDSGDTNYNPSLKS
13413817     QVHLEESGPGLVKPSETLSLTCNVSGDPRGSDNYYWGWIRQPLGKQPEWIGYVHDSGDTNYNPSLKS
15489258     RLHLEESGPDLVKPSETLSLTCNVSGGPVRGVSNYWSWIRQPLGKQPEWIGYVHDSGDTNYNPSLKS
18567397     QVHLQESGPGLVKPSETLSLTCNVSGGPFVRGSNYWSWIRQPSGKAPEWIGYVHDSGDTNYNPSLKS
              ::*:****.************.***        **.***** **  *****:: **.*********

                                                HCDR3
IGHV4-59     RVTISVDTSKNQFSLKLSSVTAADTAVYYCAR-----------------------------------
IGHV4-61     RVTISVDTSKNQFSLKLSSVTAADTAVYYCAR-----------------------------------
PGT122HC     RVHLSLDKSKNLVSLRLTGVTAADSAIYYCATTKHGRRIYGVVAFKEWFTYFYMDVWGKGTSVTVSS
14297408     RVHLSLDKSKNLVSLRLTGVTAADSAIYYCATAKHGRRIYGVVAFKEWFTYFYMDVWGKGTSVTVSS
13413817     RVHLSLDKSKNLVSLRLTGVTAADSAIYYCATTKHGRRIYGVVAFKEWFTYFYMDVWGKGTSVTVSS
15489258     RVHLSVDKSKNLVSLRLTGVTAADSAIYYCATTKHGRRIYGVVAFKEWFTYFYMDVWGKGTSVTVSS
18567397     RVHLSLDKSKKPLSLRLTGVTAADSAIYYCATTKHGRRIYGVVAFKEWFTYFYMDVWGKGTSVTVSS
             ** :*:*.**: .**:*:.*****:*:****


                                   HCDR1                          HCDR2
IGHV4-59     QVQLQESGPGLVKPSETLSLTCTVSGGSISS--YYWSWIRQPPGKGLEWIGYIYYSGSTNYNPSLKS
IGHV4-61     QVQLQESGPGLVKPSETLSLTCTVSGGSVSSGSYYWSWIRQPPGKGLEWIGYIYYSGSTNYNPSLKS
PGT124HC     QVQLQESGPGLVRPSETLSVTCIVSGGSISN--YYWTWIRQSPGKGLEWIGYISDRETTTYNPSLNS
9190560      QVQLQESGPGLVKPSETLSVTCTVSWGSMNNGTYDRSWIRQSPGKGLEWIGYVSDRAISDYNPSLKS
             ************:******:** ** **:..  *  :****.**********:     : *****:*

                                                HCDR3
IGHV4-59     RVTISVDTSKNQFSLKLSSVTAADTAVYYCAR-----------------------------------
IGHV4-61     RVTISVDTSKNQFSLKLSSVTAADTAVYYCAR-----------------------------------
PGT124HC     RAVISRDTSKNQLSLQLRSVTTADTAIYFCATARRGQRIYGVVSFGEFFYYYYMDVWGKGTAVTVSS
9190560      RVVISRDTSKNQLSLKLNSVTLADTAVYYCATARRGQRIYGEVAFGEFFYYYSMDVWGKGTAVTVSS
             *..** ******:**:* *** ****:*:**
```

FIGURE S4

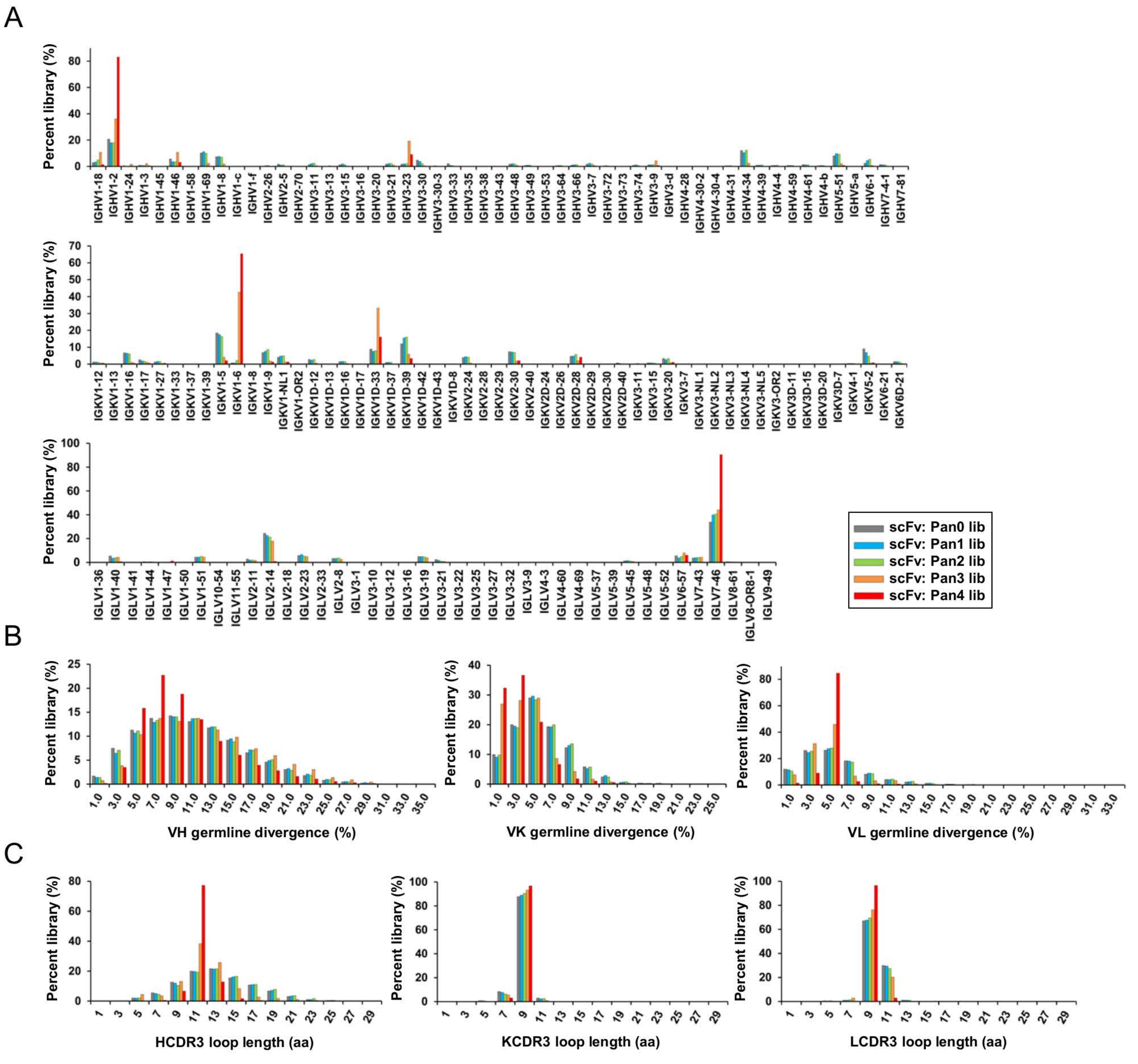

A
Percent library (%)
IGHV1-18 IGHV1-2 IGHV1-24 IGHV1-3 IGHV1-45 IGHV1-46 IGHV1-58 IGHV1-69 IGHV1-8 IGHV1-c IGHV1-f IGHV2-26 IGHV2-5 IGHV2-70 IGHV3-11 IGHV3-13 IGHV3-15 IGHV3-16 IGHV3-20 IGHV3-21 IGHV3-23 IGHV3-30 IGHV3-30-3 IGHV3-33 IGHV3-35 IGHV3-38 IGHV3-43 IGHV3-48 IGHV3-49 IGHV3-53 IGHV3-64 IGHV3-66 IGHV3-7 IGHV3-72 IGHV3-73 IGHV3-74 IGHV3-9 IGHV3-d IGHV4-28 IGHV4-30-2 IGHV4-30-4 IGHV4-31 IGHV4-34 IGHV4-39 IGHV4-4 IGHV4-59 IGHV4-61 IGHV4-b IGHV5-51 IGHV5-a IGHV6-1 IGHV7-4-1 IGHV7-81
Percent library (%)
IGKV1-12 IGKV1-13 IGKV1-16 IGKV1-17 IGKV1-27 IGKV1-33 IGKV1-37 IGKV1-39 IGKV1-5 IGKV1-6 IGKV1-8 IGKV1-9 IGKV1-NL1 IGKV1-OR2 IGKV1D-12 IGKV1D-13 IGKV1D-16 IGKV1D-17 IGKV1D-33 IGKV1D-37 IGKV1D-39 IGKV1D-42 IGKV1D-43 IGKV1D-8 IGKV2-24 IGKV2-28 IGKV2-29 IGKV2-30 IGKV2-40 IGKV2D-24 IGKV2D-26 IGKV2D-28 IGKV2D-29 IGKV2D-30 IGKV2D-40 IGKV3-11 IGKV3-15 IGKV3-20 IGKV3-7 IGKV3-NL1 IGKV3-NL2 IGKV3-NL3 IGKV3-NL4 IGKV3-NL5 IGKV3-OR2 IGKV3D-11 IGKV3D-15 IGKV3D-20 IGKV3D-7 IGKV4-1 IGKV5-2 IGKV6-21 IGKV6D-21
Percent library (%)
IGLV1-36 IGLV1-40 IGLV1-41 IGLV1-44 IGLV1-47 IGLV1-50 IGLV1-51 IGLV10-54 IGLV11-55 IGLV2-11 IGLV2-14 IGLV2-18 IGLV2-23 IGLV2-33 IGLV2-8 IGLV3-1 IGLV3-10 IGLV3-12 IGLV3-16 IGLV3-19 IGLV3-21 IGLV3-22 IGLV3-25 IGLV3-27 IGLV3-32 IGLV3-9 IGLV4-3 IGLV4-60 IGLV4-69 IGLV5-37 IGLV5-39 IGLV5-45 IGLV5-48 IGLV5-52 IGLV6-57 IGLV7-43 IGLV7-46 IGLV8-61 IGLV8-OR8-1 IGLV9-49
scFv: Pan0 lib
scFv: Pan1 lib
scFv: Pan2 lib
scFv: Pan3 lib
scFv: Pan4 lib
B
Percent library (%)
VH germline divergence (%)
VK germline divergence (%)
VL germline divergence (%)
C
Percent library (%)
HCDR3 loop length (aa)
KCDR3 loop length (aa)
LCDR3 loop length (aa)

FIGURE S5

A

Representative scFv/mAb clones identified by H/L-paired, CDR3-based clustering analysis [a]

Clone	HC name	VH identity	HCDR3 (aa)	Size	LC name	LCDR3 (aa)	Size
VAb_{d17}-1	V1P4H1	97.3%	GWGLRGQPDGY	12710	V1P4L1	LLSFGGAWV	12149
						LLAYSGAVV	100
						SLSSNGPVL	25
VAb_{d17}-2	V1P4H2	94.9%	GGLLPPHFDY	1565	V1P4L1	LLSFGGAWV	620
VAb_{d17}-3					V1P4L2	QTYDRSNVV	542
						QSYDRNTVI	178
VAb_{d17}-4	V1P4H3	94.3%	AHYDSSTWGGFAY	428	V1P4L1	LLSFGGAWV	237
						AAWDDSLSGWV	122
						QSYDSSLRTVV	13
VAb_{d17}-5	V1P4H4	97.3%	EEYRGDHDAFDI	301	V1P4K3	LQDYNYPYT	111
					V1P4L1	LLSFGGAWV	106
						LQDYNFPLT	11
VAb_{d17}-6	V1P4H5	99.7%	LYGSGSYYDY	129	V1P4L1	LLSFGGAWV	89
						LLAYSGAVV	20

[a] Listed items include scFv/mAb clone name, heavy chain (HC) name, VH germline gene identity (%), HCDR3 sequence, HC cluster size, light chain (LC) name, LCDR3 sequence, and LC cluster size.

B

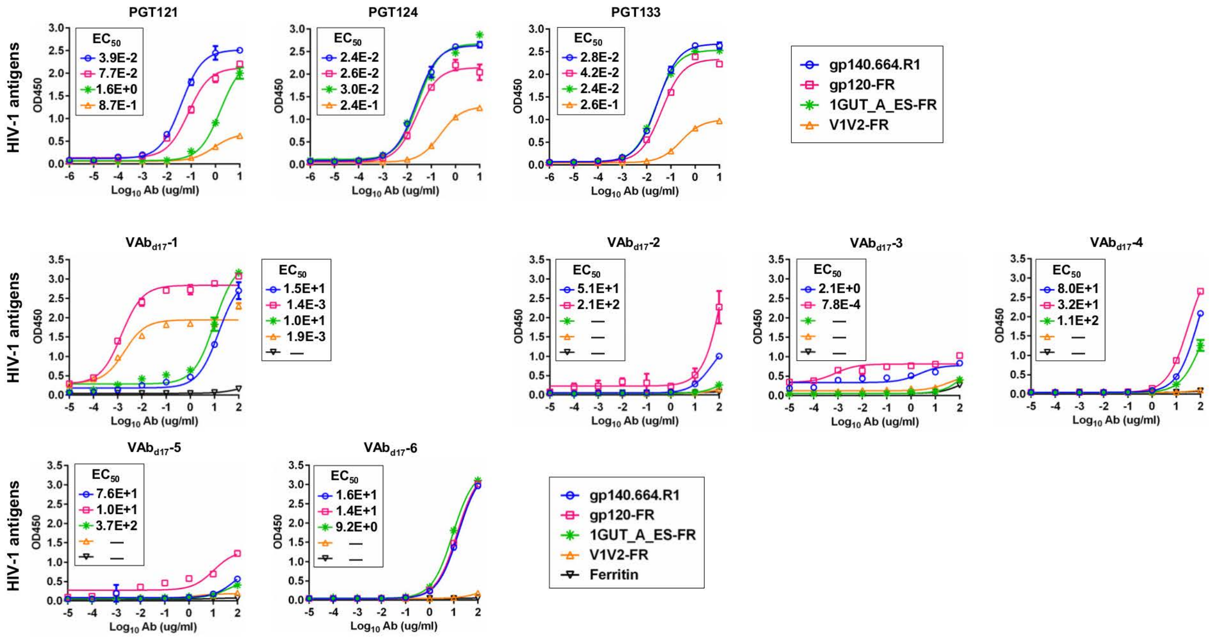

FIGURE S6

A

```
                               HCDR1                     HCDR2                                                   HCDR3
Pan0  IGHV4-34 QVQLQQWGAGLLKPSETLSLTCAVYGGSFSGYYWSWIRQPPGKGLEWIGEINHSGSTNYNPSLKSRVTISVDTSKNQFSLKLSSVTAADTAVYYCAR------------------
      107193   QVQLQQWGAGLLKPSETLSLTCAVYGGSFSGYYWSWIRQPPGKGLEWIGEINHSGSTNYNPSLKSRVTISVDTSKNQFSLKLSSVTAADTAIYFCAT [PGT124 HCDR3] WGKGTLVTVSS
               ********************************************************************************************:*:**

                               HCDR1                        HCDR2                                                       HCDR3
Pan2  IGHV4-59 QVQLQESGPGLVKPSETLSLTCTVSGGSISSYYWSWIRQPPGKGLEWIGYIYYSG-------STNYNPSLKSRVTISVDTSKNQFSLKLSSVTAADTAVYYCAR-----------
      PGT124HC QVQLQESGPGLVRPSETLSVTCIVSGGSISNYYWTWIRQSPGKGLEWIGYISDRE-------TTTYNPSLNSRAVISRDTSKNQLSLQLRSVTTADTAIYFCAT [PGT124 WGKG
      9059     QLQLQESGPGLVKPSGTLSLTCTVSGGSLSSHYWTWIRQPPGRGLEYIGYISYSGSINYNSGSTNYNPSLKSRVTISVDTSKNQFSLNMTSVTAADTATYFCAT  HCDR3] WGKG
               *:**********:** ***:** *****:*.:**:****.**:***:****       :*.*****:**..** ******:**:: ***:**** *:**

      IGHV4-59 -------
      PGT124HC TAVTVSS
      9059     TLVTVSS
```

PGT124 HCDR3: ARRGQRIYGVVSFGEFFYYYYMDV

B

```
                               LCDR1                 LCDR2                                                 LCDR3
Pan1  IGLV3-21 SYVLTQPPSVSVAPGKTARITCGGNNIGSKSVHWYQQKPGQAPVLVIYYDSDRPSGIPERFSGS---NSGNTATLTISRVEAGDEADYYCQVWDSSSDHP-----------
      PGT124LC SYVS----PLSVALGETARISCGRQALGSRAVQWYQHKPGQAPILLIYNNQDRPSGIPERFSGTPDINFGTTATLTISGVEVGDEADYYCHMWDSRSGFSWSFGGATRLTVL
      102843   SYVT----FVSVAPGQTARITWWGNNIGSKSVHWYQQKPGQAPVLVVYDDSDRPSGIPERFSGS---NSGNTATLTISRVEAGDEADYYCHMWDSRSGFSWSFGGGTKVTVL
      93072    SYVA----FVSVAPGQTAKITCGGNNIGSKSVHWYQQKPGQAPVLVVYGDSDRPSGIPERFSGS---NSGTTATLTISRVEAGDEADYYCHMWDSRSGFSWSFGGGTELTVL
      86916    SYVS----DISVAPGQTATMTCGGTNIGSQSVHWFQQKPGQAPVLVVYDDDHRPSGIPEQFSGS---KSGNTASLTISRVAAADEADYYCHMWDSRSGFSWSFGGGTELTVL
               ***         :*** *:** ::      :**::*:*:*:******:*::* :..*******:***:    : *.**:**** * ..*******::*** *...

      IGLV3-21 SYVLTQPPSVSVAPGKTARITCGGNNIGSKSVHWYQQKPGQAPVLVIYYDSDRPSGIPERFSGS---NSGNTATLTISRVEAGDEADYYCQVWDSSSDHP-----------
      PGT133LC S-LNP----LSLAPGATAKIPCGERSRGSRAVQWYQQKPGQAPTLIIYNNQDRPAGVSERFSGNPDVAIGVTATLTISRVEVGDEADYYCHYWDSRSPISWIFGGGTQLTVL
      1437     SYVSD----ISVAPGQTARITCGGNNIGSKSVHWYQRKPGQAPVLVVYDDSDRPSGIPERFSGS---NSGNTATLTISRVEAGDEADYYCHYWDSRSPISWIFGGGTKLTVL
      2953     SYVSP----LSVAPGQTARITCGGNNIESKSVHWLQQKPGQAPVLVLYDDSDRPSGIPERFSGS---NSENTATLTITRAEAGDEADYYCHYWDSRSPISWIFGGGTKVTVL
      72793    SYVTF----VSVAPGKTATITCGANKIGTKSVHWYQQMTGQAPVLVMFDDNERPSGIPERLSGS---NSGDTAALTISRVEAGDEADYYCHYWDSRSPISWIFGGGTKLTVL
      29001    SYVSP----LSVAPGQTATMTCGGPNIRSQSVHWFQQRPGQAPVLVVYDDDHRPSGIPEQFSGS---KSGNTASLTISRVAAADEADYYCHYWDSRSPISWIFGGGTQLTVL
                 :         :*:*** ** :.**  .  :::*:* *: .****.*::: :..**:*:.*::**.        **:***:*. ..*******: *** *   .
```
